# A microprotein N1DARP encoded by LINC00261 promotes Notch1 intracellular domain (N1ICD) degradation via disrupting USP10-N1ICD interaction to inhibit chemoresistance in Notch1-hyperactivated pancreatic cancer

**DOI:** 10.1038/s41421-023-00592-6

**Published:** 2023-09-15

**Authors:** Shuyu Zhai, Jiewei Lin, Yuchen Ji, Ronghao Zhang, Zehui Zhang, Yizhi Cao, Yang Liu, Xiaomei Tang, Jia Liu, Pengyi Liu, Jiayu Lin, Fanlu Li, Hongzhe Li, Yusheng Shi, Da Fu, Xiaxing Deng, Baiyong Shen

**Affiliations:** 1 Department of General Surgery, Pancreatic Disease Center, Ruijin Hospital, Shanghai Jiaotong University School of Medicine, Shanghai, China; 2 Research Institute of Pancreatic Diseases, Shanghai Jiaotong University School of Medicine, Shanghai, China; 3 State Key Laboratory of Oncogenes and Related Genes, Institute of Translational Medicine, Shanghai Jiaotong University, Shanghai, China; 4 Department of Thoracic Surgery, Shanghai Pulmonary Hospital, Tongji University School of Medicine, Shanghai, China

**Keywords:** Pancreatic cancer, Targeted therapies, Cancer therapeutic resistance, Ubiquitylation, Long non-coding RNAs

## Abstract

The extensively activated Notch signaling pathway in pancreatic cancer cells is important in carcinogenesis, chemoresistance, and recurrence. Targeting this pathway is a promising therapeutic strategy for pancreatic cancer; however, few successful approaches have been reported, and currently used molecular inhibitors of this pathway exhibit limited clinical benefits. In this study, we identified a previously uncharacterized microprotein, Notch1 degradation-associated regulatory polypeptide (N1DARP), encoded by *LINC00261*. N1DARP knockout accelerated tumor progression and enhanced stem cell properties in pancreatic cancer organoids and LSL-Kras, LSL-Trp53, and Pdx1-Cre (KPC) mice. Mechanistically, N1DARP suppressed canonical and non-canonical Notch1 pathways by competitively disrupting the interaction between N1ICD and ubiquitin-specific peptidase 10 (USP10), thereby promoting K11- and K48-linked polyubiquitination of N1ICD. To evaluate the therapeutic potential of N1DARP, we designed a cell-penetrating stapled peptide, SAH-mAH2-5, with a helical structure similar to that of N1DARP that confers remarkable physicochemical stability. SAH-mAH2-5 interacted with and promoted the proteasome-mediated degradation of N1ICD. SAH-mAH2-5 injection provided substantial therapeutic benefits with limited off-target and systemic adverse effects in Notch1-activated pancreatic cancer models. Taken together, these findings confirm that N1DARP acts as a tumor suppressor and chemosensitizer by regulating USP10-Notch1 oncogenic signaling, and suggest a promising therapeutic strategy targeting the N1DARP–N1ICD interaction in Notch1-activated pancreatic cancer.

## Introduction

Pancreatic cancer is one of the most malignant digestive cancers and has the worst prognosis compared with other major malignancies, with a 5-year survival rate of < 10%^1^. Only 20% of patients with pancreatic cancer are eligible for surgery, whereas the others have to be treated with chemotherapy^2^. Pancreatic ductal adenocarcinoma (PDAC) is known for its refractory resistance to standard chemotherapy, which can be attributed to its inherent intratumoral heterogeneity and highly desmoplastic tumor microenvironment^3^. Recent evidence has supported the use of highly conserved signaling pathways that are consistently activated in pancreatic cancer, such as the Notch, Hedgehog, and Wnt signaling pathways, to identify novel biomarkers and therapeutic targets^4,5^.

The Notch pathway is active in a broad spectrum of human cancers, including breast, colorectal, lung, prostate, glioblastoma, and pancreatic cancer^6^. Notch signaling is positively associated with pancreatic tumor initiation, progression, and drug resistance in both cell lines and genetic mutant mouse models^7,8^. In preclinical models, pharmacological inhibition of Notch signaling by small molecules designed to affect HES1 transcriptional function (JI051 and JI130)^9^ and natural agents targeting the Notch pathway, such as curcumin, genistein, quercetin, and sulforaphane, slowed tumor proliferation and invasion, induced intratumor apoptosis, and potentiated chemotherapy^10–12^. Notably, γ-secretase inhibitors that block Notch cleavage, including PF-03084014, MK-0752, and RO4929097, have been administered in clinical studies to treat metastatic pancreatic cancers. Unfortunately, no significant remission was observed^13–15^, possibly due to dual or pan-Notch inhibition of Notch1 and other family members, leading to the compensatory activation of other signaling pathways^16,17^. This suggests that specific targeting of individual Notch family members or other ligands, such as antibodies or protein subunits that block protein–protein interactions, is a promising approach to disrupt Notch signaling. Recently, a phase II study focusing on metastatic pancreatic cancer treated with tarextumab, a fully human IgG2 antibody that inhibits Notch2/3 receptors, in combination with gemcitabine (GEM) and paclitaxel, showed remarkable remission and tolerance among patients, indicating a promising future for therapies targeting specific Notch family members^18^.

Notch is a family of short-lived proteins that undergoes rapid degradation mainly through the ubiquitin-proteasome system, with variable components that positively or negatively regulate Notch1 stability^6,19^. This suggests that promoting ubiquitin-mediated degradation of Notch family members could be a promising strategy for targeting Notch-activated cancers. USP10, a deubiquitinating enzyme with a highly conserved sequence, is a negative p53 regulator involved in tumorigenesis^20–22^. USP10 confers chemoresistance in cancer cells by deubiquitinating multiple proteins^23–25^. N1ICD plays a vital role in USP10-mediated biological processes such as cell development, angiogenesis, and cardiac dysfunction^26,27^. Therefore, targeting the USP10–N1ICD interaction could be a potential antitumor strategy for Notch1-hyperactivated cancers.

Long non-coding RNAs (lncRNAs) function as signals, decoys, guides, and scaffolds^28,29^. Recent evidence has demonstrated that functional peptides that play pivotal roles in multiple biological processes are encoded by short open reading frames (ORFs) in lncRNAs^30–34^. Nevertheless, few lncRNA-encoded peptides and their roles in tumorigenesis and tumor progression have been reported in pancreatic cancer. Here, we identified a previously uncharacterized polypeptide, N1DARP, encoded by *LINC00261*, through RNA sequencing and ribosome profiling, as a novel tumor suppressor and chemotherapy sensitizer for use in pancreatic cancer therapy, which acts by interfering with USP10–N1ICD interaction. Our study also evaluated the therapeutic potential of targeting N1DARP using a modified stapled peptide derived from N1DARP, SAH-mAH2-5, which perturbs the USP10–N1ICD interaction and suppresses tumor growth in Notch1-activated pancreatic cancer, thus providing insights into how the N1DARP–N1ICD interaction could be targeted for the development of precise and individualized pancreatic cancer therapy.

## Results

### Identification of functional peptides encoded by *LINC00261*

**Fig. 1:**
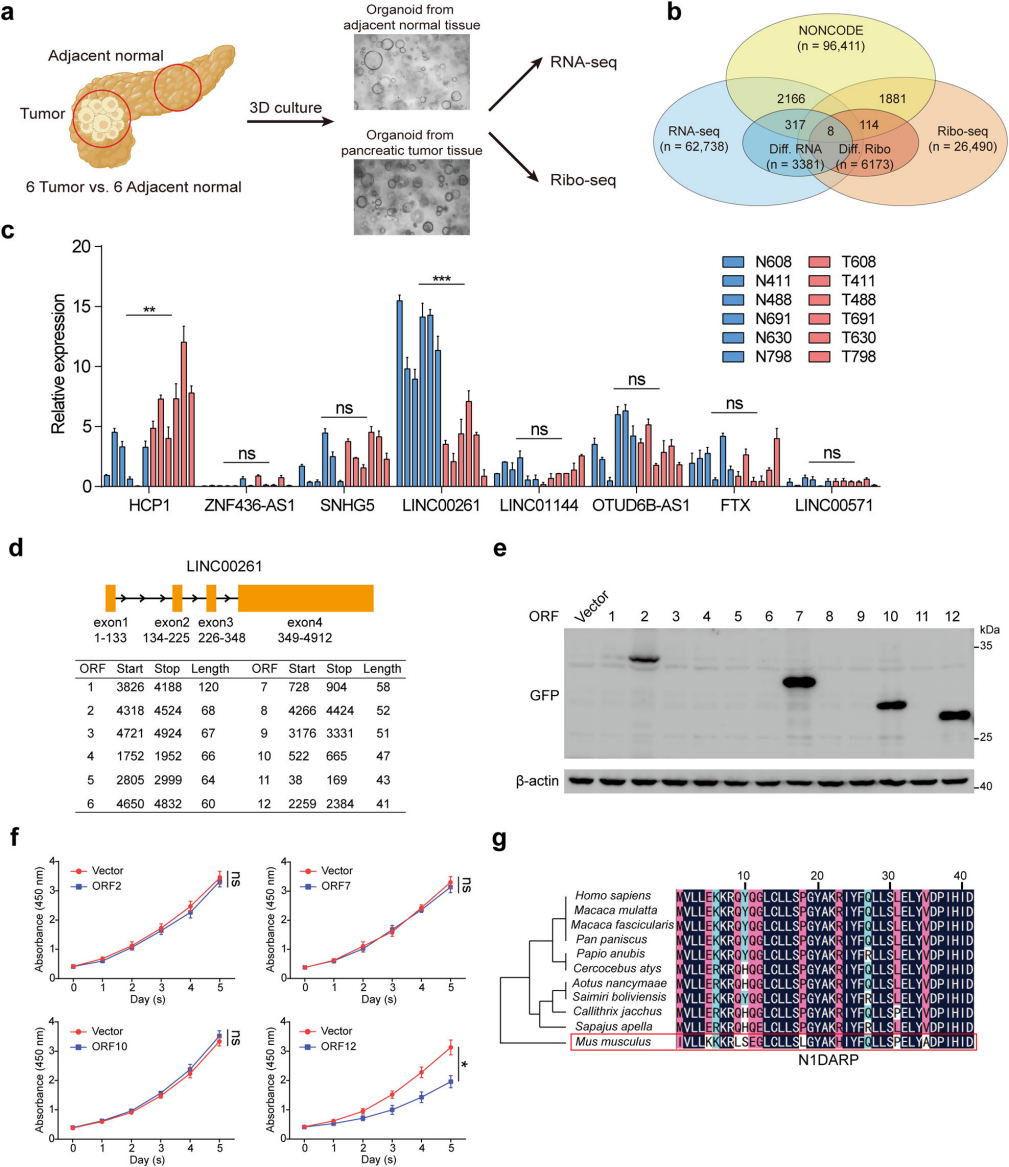
N1DARP is a functional 41 amino acid peptide encoded by *LINC00261*. **a** Preparation of mRNA sequencing and ribosome profiling using patient-derived organoid model. **b** Intersection of differentially expressed and translated lncRNAs annotated by NONCODE in pancreatic cancer and normal pancreatic cells. **c** Expression of eight lncRNAs in six pairs of pancreatic cancer and normal pancreas organoids. **d** ORFs distributed in the positive strand of *LINC00261* exons with more than 40 amino acids obtained from ORFfinder. **e** GFP-tagged polypeptides encoded by *LINC00261* as detected by western blotting assay using HEK293T. **f** Cell viability detected by CCK8 assay using Panc1 transfected with coding ORFs. **g** Alignment analysis of N1DARP amino acid sequence in primates and mice. The data are presented as the mean ± SD of three independent experiments. ns no significance; *P < 0.05, **P < 0.01, ***P < 0.001 by one-way ANOVA (**c**, **f**).

To identify potentially functional and coding lncRNAs in pancreatic cancer cells, we performed RNA sequencing and ribosome profiling of normal pancreatic organoids and pancreatic tumor organoids derived from adjacent normal pancreatic tissue and pancreatic cancer tissue from six pairs of patients diagnosed with PDAC (Fig. 1a). We established normal and tumor organoids for RNA sequencing and ribosome profiling because ribosome profiling often requires a large number of cells to provide reliable results. However, pancreatic tumor tissue contains a dominant proportion of fibroblasts and collagen (up to 80%, as reported)^35^, while tumor cells rarely exist. Therefore, we extracted tumor cells from tumor tissues, established corresponding tumor organoids to maintain their genetic characteristics, and allowed them to propagate to adequate cell numbers for ribosome profiling. For comparison, we used the same group of tumor organoids for RNA sequencing. We identified 3381 differentially expressed RNAs, of which 325 were differentially expressed lncRNAs (DElncRs) (Fig. 1b). Among these DElncRs, 84 were downregulated and 241 were upregulated (Supplementary Fig. S1a). Similarly, we identified 6173 differentially translated RNAs and 122 differentially translated lncRNAs (DTlncRs) using Ribo-seq in accordance with the criteria that unique junction reads were identified in more than six samples, and more than 100 total junction reads were observed (Fig. 1b and Supplementary Fig. S1b, c). By cross-referencing DTlncRs to DElncRs, we obtained eight candidate genes that were both differentially expressed and translated in pancreatic cancer (Fig. 1b and Supplementary Fig. S1c). Next, we obtained expression data and follow-up information from the Cancer Genome Atlas (TCGA) database and found that only *LINC00261* exhibited both differential expression and prognosis (Supplementary Fig. S1d, e). Following biological validation, we found that *LINC00261* showed the highest differential expression between pancreatic cancer and normal pancreatic epithelial cells (Fig. 1c and Supplementary Fig. S1f). In addition, by screening the results of mass spectrometry from our previously published data^36^, we found that *LINC00261* interacted with RPS6, which has been reported as a key structural constituent of the 40S ribosomal subunit that mediates translation initiation of mRNAs with poly A tails, by interacting directly with the 5′-m7 GpppG cap-binding complexes^37,38^. Consistent with our results, a recent study revealed that *LINC00261* was capable of translation during pancreatic exocrine cell development^39^. In addition, as evaluated by the online tool CPC2^40^, *LINC00261* had a higher coding probability than other verifiably translatable lncRNAs (Supplementary Fig. S2a)^30,31^. These results suggest that *LINC00261* has a coding capacity.

Next, to validate the coding capacity of *LINC00261*, we constructed 12 GFP-tagged plasmids, which contained ORFs in *LINC00261*, obtained using ORFfinder; these were located in the positive strand with more than 40 amino acids that have been considered as functional peptides (Fig. 1d)^41^. The results showed that ORF2, ORF7, ORF10, and ORF12 could be translated (Fig. 1e). To investigate the potential biological functions of these ORFs, we performed a CCK8 assay which showed that only ORF12 overexpression inhibited the proliferation of pancreatic cancer cells (Fig. 1f). Moreover, alignment analysis demonstrated that the amino acid sequence of ORF12 was evolutionarily conserved in primates and slightly less conserved in mice, which facilitated the genetic editing of N1DARP in mice in subsequent experiments (Fig. 1g). These findings suggest that ORF12 encodes a previously unrecognized 41-amino acid peptide, N1DARP, with a potential tumor-suppressive function. To further verify the presence of N1DARP in pancreatic cancer, we generated, apart from the wild-type GFP (GFPwt) constructed above, a start codon mutant GFP (GFPmut) and start codon wild-type or mutant ORF12 tagged with GFPmut and FLAG labels (ORF12-GFPmut, ORF12mut-GFPmut, ORF12-Flag, and ORF12mut-Flag; Supplementary Fig. S2b) and transfected into Panc1 for 48 h. We found that ORF12 tagged with either GFP or Flag could be translated into microproteins through fluorescent microscopy, immunofluorescent assay, and western blotting assay, whereas ORF12mut could not (Supplementary Fig. S2c–e), suggesting that N1DARP was present in pancreatic cancer cells. To determine whether N1DARP is endogenously expressed, we generated a polyclonal rabbit N1DARP antibody. The specificity of the antibody was verified (Supplementary Fig. S2f). The endogenous presence of N1DARP in the cytoplasm was detected in Panc1 cells using immunofluorescence microscopy (Supplementary Fig. S2g). In addition, enriched N1DARP was obtained from Panc1 cells using immunoprecipitation with an N1DARP antibody and then subjected to mass spectrometry to further prove the existence of N1DARP in pancreatic cancer cells (Supplementary Fig. S2h).

### N1DARP suppresses proliferation and stemness in pancreatic cancer cells

N1DARP expression was measured in pancreatic cancer cells and compared with its normal counterparts. The expression of N1DARP was reduced in cancer cells and tissues (Supplementary Fig. S3a–c), and its low expression was associated with a worse prognosis (Supplementary Fig. S3d). N1DARP expression was also negatively correlated with the expression levels of Ki-67 and SOX-2 (Supplementary Fig. S3e). Clinically, the protein expression of N1DARP as measured by immunohistochemistry assay was negatively correlated with the pathological and T stages of patients with pancreatic cancer (Supplementary Table S1). These results suggest a tumor suppressor role for N1DARP. Next, to further investigate the tumor-suppressive function of N1DARP, we established *LINC00261* and *N1DARP* overexpression models by stably transfecting lentiviruses containing full-length *LINC00261* and *N1DARP* into Capan1, and constructed a Crispr/Cas9-mediated *LINC00261* and *N1DARP* knockout model by deleting full-length *LINC00261* and partial exon 4 of *LINC00261* containing full-length *N1DARP* in Panc1 (Supplementary Fig. S3f). We constructed these two cell line models because, as shown in Supplementary Fig. S3a, the Capan1 cell line exhibited the lowest expression, whereas Panc1 cells showed the highest expression level of N1DARP. The transfection efficiency was assessed by western blotting assay (Supplementary Fig. S3g).

**Fig. 2:**
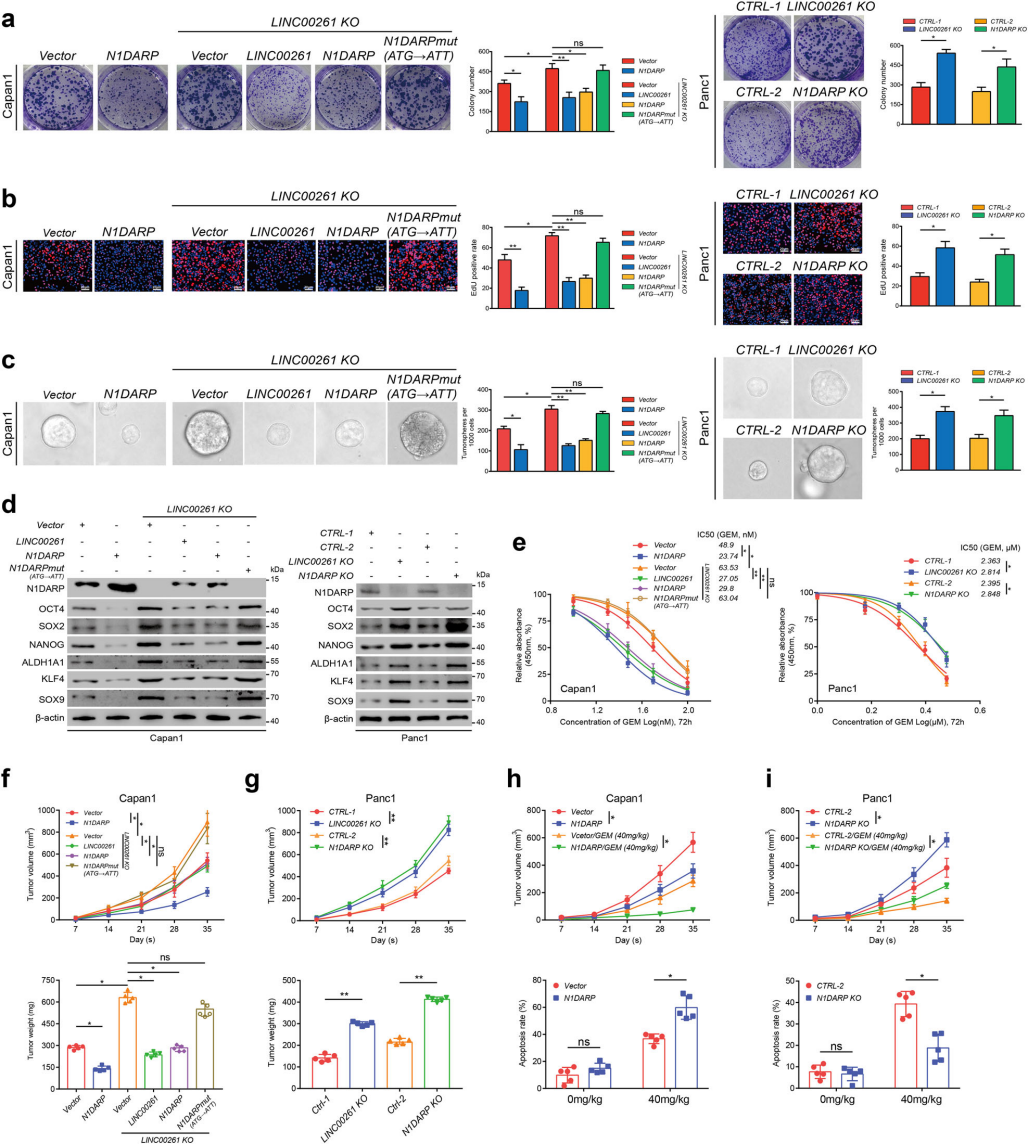
Overexpression of N1DARP suppresses proliferation and stem cell properties of pancreatic cancer cells. **a**, **b** Cell proliferation capacity was evaluated by (**a**) CCK8 assay and (**b**) EdU assay using wild-type or *LINC00261* knocked-out Capan1 transfected with N1DARPwt or N1DARPmut plasmid, and Panc1 with *N1DARP* or *LINC00261* knockout. **c**, **d** Stem cell properties were assessed by sphere formation assay (**c**) and western blotting assay (**d**) for detecting stemness biomarkers using the same grouped Capan1 and Panc1 cells mentioned above. **e** IC_50_ assay used to detect chemosensitivity to GEM in wild-type or *LINC00261* knocked-out Capan1 transfected with N1DARPwt or N1DARPmut plasmid and in Panc1 cells with *N1DARP* or *LINC00261* knockout. **f**, **g** Tumor volume and weight of a subcutaneously injected xenograft model using wild-type or *LINC00261* knocked-out Capan1 transfected with N1DARPwt or N1DARPmut plasmid, and Panc1 with *N1DARP* or *LINC00261* knockout. **h**, **i** Tumor volume and apoptosis rate detected by flow cytometry of a subcutaneously injected xenograft model treated with GEM (40 mg/kg, biweekly) using Capan1 overexpressing N1DARP or Panc1 with *N1DARP* knockout. Data are presented as mean ± SD of three independent experiments. ns not significant; *P < 0.05, **P < 0.01 by Student’s *t*-test (**a**–**c**, **f**–**g** for *t*umor weight, and **h**, **i** for apoptosis rate); ns not significant; *P < 0.05, **P < 0.01 by one-way ANOVA (**e**, **f**–**i** for tumor volume). Scale bars, 20 μm (**b**).

Overexpression of *N1DARP* in Capan1 cells resulted in reduced growth and stem cell properties and enhanced sensitivity to GEM, while *N1DARP* knockout in Panc1 enhanced tumorigenesis and suppressed chemosensitivity (Fig. 2a–e). Using *LINC00261* knockout Capan1 transfected with the same dose of FLAG-tagged *LINC00261* and *N1DARP* plasmid, or Panc1 with *LINC00261* or *N1DARP* knockout, we found that N1DARP showed effects comparable to *LINC00261* in regulating cell proliferation, stemness traits, and chemosensitivity (Fig. 2a–e). In addition, *LINC00261* knockout Capan1 transfected with a FLAG-tagged mutation of the start codon of *N1DARP* (ATG → ATT), as demonstrated in Supplementary Fig. S2b, showed almost no alteration in cell proliferation, stemness, or chemosensitivity (Fig. 2a–e). Furthermore, subcutaneously injected tumors in nude mice using Capan1 cells with ectopically expressed *N1DARP* showed retarded tumor growth and enhanced chemosensitivity, whereas *N1DARP* knockout in Panc1 accelerated tumor growth and reduced chemosensitivity in vivo (Fig. 2f–i). N1DARP exhibited antitumor effects similar to those of *LINC00261* (Fig. 2f–i). Concordantly, the start codon mutation of N1DARP showed no biological function in vivo (Fig. 2f–i). In addition, to determine the role of N1DARP in the *LINC00261*-mediated tumor-suppressive signaling pathway, we generated two *LINC00261* overexpression cell models with or without the start codon mutant of N1DARP (LINC00261-N1DARPwt and LINC00261-N1DARPmut). Colony formation and EdU assays using Capan1 showed that N1DARP deletion partially rescued the suppressed cell proliferation induced by *LINC00261* overexpression (Supplementary Fig. S4a, b). However, sphere formation assay and western blotting assay showed that silencing N1DARP almost completely rescued *LINC00261*-induced inhibition of stem cell properties in pancreatic cancer (Supplementary Fig. S4c, d). In vivo assay demonstrated that *LINC00261*-induced retardation of tumor growth was entirely rescued by N1DARP deletion (Supplementary Fig. S4e). The above results suggest that *LINC00261* as a lncRNA could partially regulate tumor growth, while *LINC00261-*encoded N1DARP as a peptide accounted for a major proportion of the control of cell proliferation and entirely mediated the stemness traits of pancreatic cancer.

**Fig. 3:**
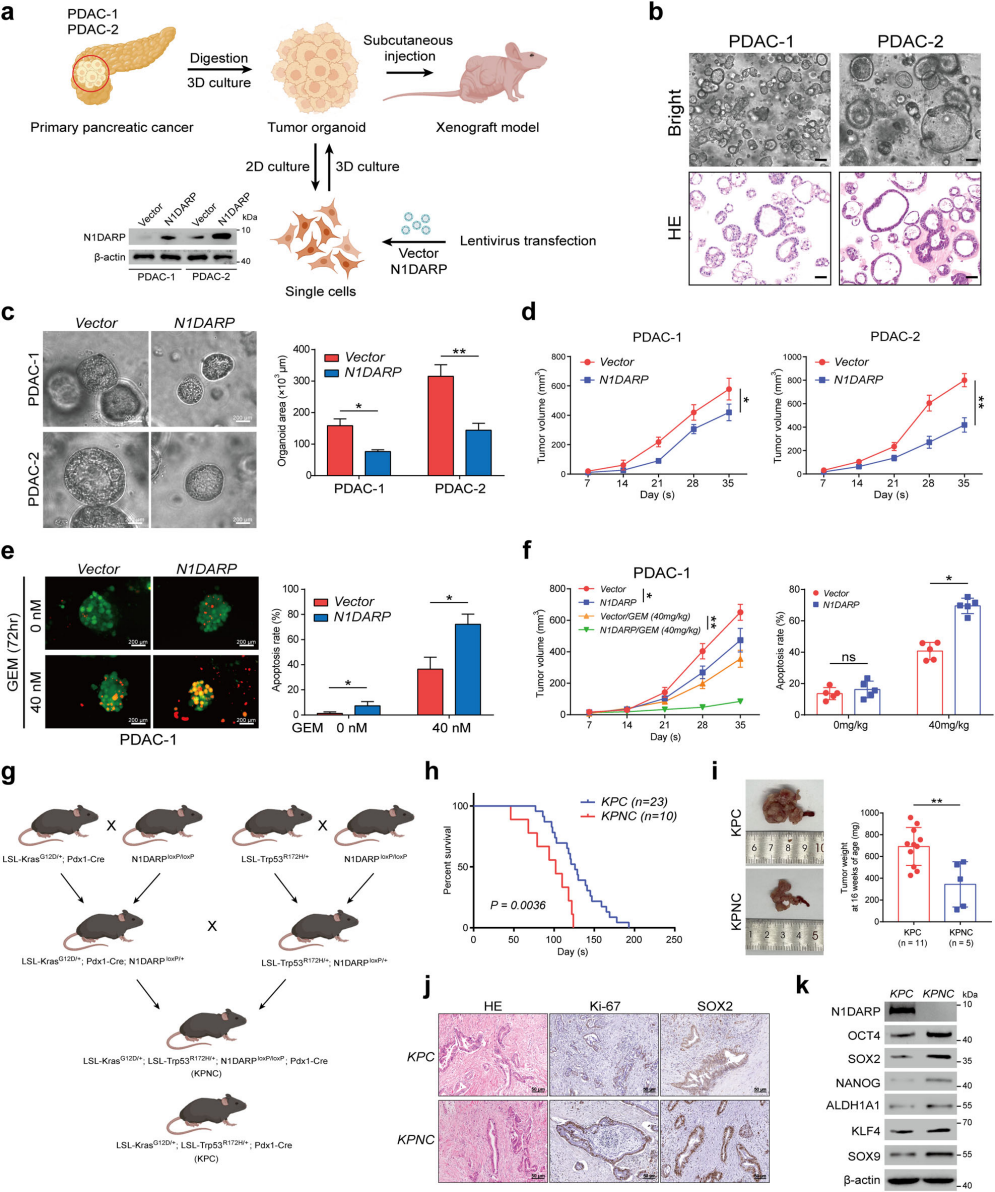
N1DARP overexpression inhibits tumorigenesis and stem cell traits of patient-derived organoids and a genetically engineered mouse model. **a** Establishment of pancreatic cancer organoids and cells transfected with vector or N1DARP through subcutaneous injection into nude mice. **b** Bright-field image and immunohistochemistry analysis of PDAC-1 and PDAC-2 showing their successful establishment. **c** Left: Microscopic inspection of organoid diameter of PDAC-1 and PDAC-2 transfected with vector or N1DARP. Right: Calculated average organoid area of PDAC-1 and PDAC-2. **d** Tumor volume of transfected PDAC-1 and PDAC-2 through subcutaneous injection of vector or N1DARP. **e** Left: Calcein-AM/PI staining visualized by fluorescence microscopy detecting apoptotic cells of PDAC-1 and PDAC-2 organoid with control or N1DARP overexpression followed by treatment with GEM for 72 h. The green signal indicates living tumor organoid, while red dots denote apoptotic cells. Right: quantified apoptotic rate of PDAC-1 and PDAC-2 with vector or N1DARP transfection treated with GEM detected by flow cytometry. **f** Tumor volume and apoptosis rate detected by flow cytometry of subcutaneously injected mice model using wild-type or N1DARP overexpressed PDAC-1 treated with GEM (40 mg/kg, biweekly). **g** Generation of KPC and KPNC mice. **h** Survival analysis with follow-up data of KPC and KPNC. **i** Tumor weight of KPC and KPNC mice at 16 weeks of age. **j** Hematoxylin and eosin (HE) staining and immunohistochemistry analysis of Ki-67 and SOX-2 using spontaneous pancreatic tumor from KPC and KPNC using western blot analysis. **k** Detection of biomarkers of stem-like properties using western blot analysis of spontaneous pancreatic tumor from KPC and KPNC. The data are presented as the mean ± SD of three independent experiments. *P < 0.05, **P < 0.01 by Student’s *t*-test (**c**, **e**, **f** for apoptosis rate; **i** for tumor weight); *P < 0.05, **P < 0.01 by one-way ANOVA (**d**, **f** for tumor volume); *P* < 0.05 was considered statistically significant by log-rank test (**h**). Scale bars, 50 μm (**b**, **j**), 200 μm (**c**, **e**).

Furthermore, we constructed and verified two distinct pancreatic cancer patient-derived organoid models (PDAC-1 and PDAC-2; Fig. 3a, b). Through microscopic inspection and in vivo subcutaneous tumor transplantation, we found that PDAC-1 and PDAC-2 with overexpressed *N1DARP* exhibited reduced growth of pancreatic cancer organoids (Fig. 3c, d). In addition, calcein-AM/PI staining followed by fluorescence microscopy confirmed that N1DARP overexpression in PDAC-1 and PDAC-2 cells increased vulnerability to GEM (Fig. 3e). Subcutaneous tumor transplantation using PDAC-1 and PDAC-2 cells transfected with N1DARP, followed by treatment with GEM, further corroborated the chemosensitizing role of N1DARP in vivo (Fig. 3f). Moreover, we generated Pdx1-Cre-mediated *N1DARP* conditional knockout mice in which *N1DARP* was specifically deleted in the pancreatic tissue. The *N1DARP* knockout mice were then crossed with congenic KPC mice to generate LSL-Kras^G12D/+^; LSL-Trp53^R172H/+^; N1DAR^PloxP/loxP^; Pdx1-Cre (KPNC) mice (Fig. 3g). KPNC mice had a shortened median survival (86.0 vs 139.5 days) and accelerated tumor progression compared with KPC mice (Fig. 3h, i). The pancreatic cancer tissues of the KPNC mice exhibited enhanced expression of Ki-67, Sox-2, and other cancer stem cell biomarkers (Fig. 3j, k). In summary, our results indicate that N1DARP is a potent tumor suppressor and chemosensitizer in pancreatic cancer that mitigates cancer stem cell characteristics and tumor formation capacity.

### N1DARP suppresses tumors through interacting with and enhancing proteosome-mediated degradation of N1ICD

**Fig. 4:**
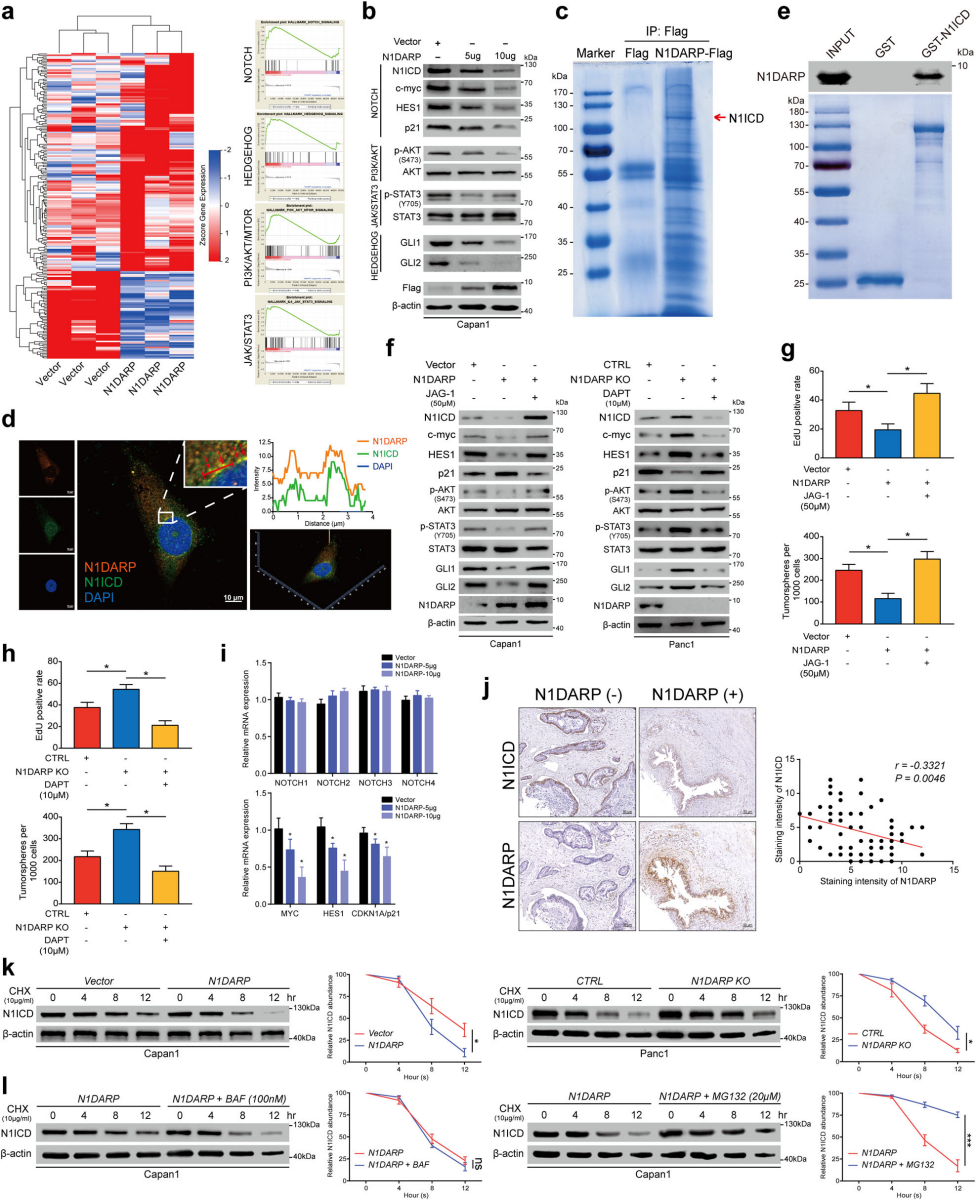
N1DARP inhibits pancreatic cancer initiation and progression by mitigating activation of the Notch signaling pathway. **a** Left: RNA sequencing analysis using Capan1 transfected with vector or N1DARP overexpression plasmid. Right: GSEA analysis demonstrating the enrichment of differentially expressed genes in Notch, Hedgehog, PI3K/AKT, and JAK-STAT3 pathways. **b** Western blotting analysis showing inhibition of Notch1 signaling and its crosstalk with other pathways in Capan1 transfected with FLAG-tagged N1DARP plasmid in a dose-dependent manner. **c** Co-IP followed by mass spectrometry revealing the N1DARP–N1ICD interaction in HEK293T. **d** Immunofluorescence confocal microscopy showing the co-localization of N1DARP and N1ICD in the cytoplasm by the simultaneous elevation of fluorescence intensity of these two proteins in Panc1 cytoplasm. The red arrows indicate the co-localization site and the red line represents fluorescence intensity measured in the right panel. **e** In vitro pulldown assay performed using purified GST and GST-tagged N1ICD fusion protein incubated with Panc1 cell lysates, followed by western blotting analysis to detect the direct interaction between N1DARP and N1ICD. **f** Western blotting analysis showing the expression of key proteins in Notch signaling and its related pathways with N1DARP overexpression or repression, followed by treatment with Notch signaling activator JAG-1 or inhibitor DAPT. **g**, **h** Cell proliferation detected by EdU assay and stem cell properties measured by tumor sphere formation assay using Capan1 (**g**) transfected with N1DARP followed by treatment with JAG-1, a Notch pathway agonist, and Panc1 (**h**) with *N1DARP* knockout following DAPT treatment, a Notch pathway inhibitor. **i** RNA expression of Notch receptors (*Notch1*, *Notch2*, *Notch3*, and *Notch4*) and Notch signaling targeted genes (*c-myc*, *HES1*, and *p21*) measured by qRT-PCR using Capan1 with vector or N1DARP overexpression. **j** Immunohistochemistry analysis using tissue microarray from seventy-five pancreatic cancer patients detecting the correlation between N1DARP and N1ICD. **k** Western blotting analysis exhibiting N1ICD remaining level at indicated time in Capan1 and Panc1 with N1DARP overexpression or knockout and treatment with CHX, a protein synthesis inhibitor. **l** Western blotting analysis exhibiting N1ICD levels remaining at indicated time after treatment with Capan1 with N1DARP overexpression and BAF, a lysosome inhibitor, and MG132, a proteosome inhibitor. The gray level was quantified using ImageJ. The data are presented as the mean ± SD of three independent experiments. *P < 0.05 by Student’s *t*-test (**g**–**i**); *P* < 0.05 was considered statistically significant by Pearson’s correlation test (*r*, Pearson’s correlation coefficient) (**j**); ns no significance; *P < 0.05, ***P < 0.001 by one-way ANOVA (**k,** **l**). Scale bars, 10 μm (**d**), 50 μm (**j**).

To reveal the downstream molecular mechanisms through which N1DARP exerts its tumor-suppressive function, we performed RNA sequencing using Capan1 transfected with a vector or N1DARP overexpression plasmid (Fig. 4a). Gene set enrichment analysis (GSEA) of the results demonstrated that the differentially expressed genes were enriched in the Notch, Hedgehog, JAK-STAT3, and PI3K-AKT signaling pathways (Fig. 4a). This bioinformatic analysis was further validated using western blotting assay in Capan1 transfected with a vector or different doses of FLAG-tagged N1DARP plasmid, where overexpressed N1DARP suppressed the activation of Notch, Hedgehog, JAK-STAT3, and PI3K-AKT pathways in a dose-dependent manner (Fig. 4b), which are well-established signaling pathways related to tumor initiation and progression of pancreatic cancer.

There are two widely accepted mechanisms by which microproteins exert their biological or pathological functions: secretion into the extracellular matrix and interactions with other proteins. After determining the mRNA sequence of N1DARP, we found no signal peptide sequences. Therefore, N1DARP likely functions by binding to other proteins. We then performed co-IP followed by mass spectrometry analysis using Panc1 cells transfected with N1DARP-Flag and found that Notch1 was at the top of the list (Fig. 4c). We verified these results by co-IP using Panc1 and Capan-1 cells and found that Notch1 was present at ~120 kDa, suggesting that this interactive protein could be the N1ICD (Supplementary Fig. S5a). In addition, fluorescence confocal microscopy revealed the co-localization of N1DARP and N1ICD in the cytosol of pancreatic cancer cells (Fig. 4d). Furthermore, a GST-tagged pull-down and Proximity Ligation Assay (PLA) using Panc1 cells demonstrated a direct interaction between N1DARP and N1ICD (Fig. 4e and Supplementary Fig. S5b). Based on these results, we presumed that N1DARP functions by interacting with and repressing N1ICD expression, leading to the suppression of Notch signaling. To test this hypothesis, we performed western blot analysis of Capan1 cells overexpressed with N1DARP and then treated them with JAG-1, a Notch signaling activator, or in Panc1 with *N1DARP* knockout and treated with DAPT, a Notch signaling inhibitor. The results showed that Notch1 signaling and its crosstalk with other signaling pathways were inhibited by elevated N1DARP but rescued by JAG-1 or activated by *N1DARP* knockout but rescued by DAPT (Fig. 4f). Consistently, N1DARP diminished tumor proliferation and sphere formation by impinging on Notch signaling (Fig. 4g, h). Taken together, these findings indicated that N1DARP suppressed tumorigenesis via the Notch1 pathway by interacting with N1ICD.

To determine how N1DARP suppresses N1ICD expression, we performed qRT-PCR to detect the mRNA expression of *Notch1*, *Notch2*, *Notch3*, and *Notch4* using N1DARP-overexpressed Capan1. We found that the mRNA levels of downstream targets of the Notch pathway, including *c-Myc*, *HES1*, and *p21*, but not Notch receptors, were inhibited by N1DARP overexpression (Fig. 4i), suggesting that N1DARP regulates N1ICD expression in a post-transcriptional manner. In addition, a tissue array from seventy-five pancreatic cancer showed that N1DARP protein levels were negatively correlated with N1ICD protein levels (Fig. 4j), supporting the above suggestion. To investigate how N1DARP post-transcriptionally inhibits N1ICD expression, we measured N1ICD protein level every 4 h in Capan1 cells with N1DARP overexpression or Panc1 with *N1DARP* knockout after treatment with cycloheximide (CHX), a protein synthesis inhibitor. Western blotting assay showed attenuated N1DARP overexpression, whereas *N1DARP* knockout strengthened the stability of N1ICD (Fig. 4k). Furthermore, the effect of N1DARP on N1ICD stability was abrogated by the proteasome inhibitor (MG132), but not by the lysosomal inhibitor (bafilomycin, BAF), indicating that N1DARP promoted N1ICD degradation through the ubiquitin-proteasome pathway (Fig. 4l).

**Fig. 5:**
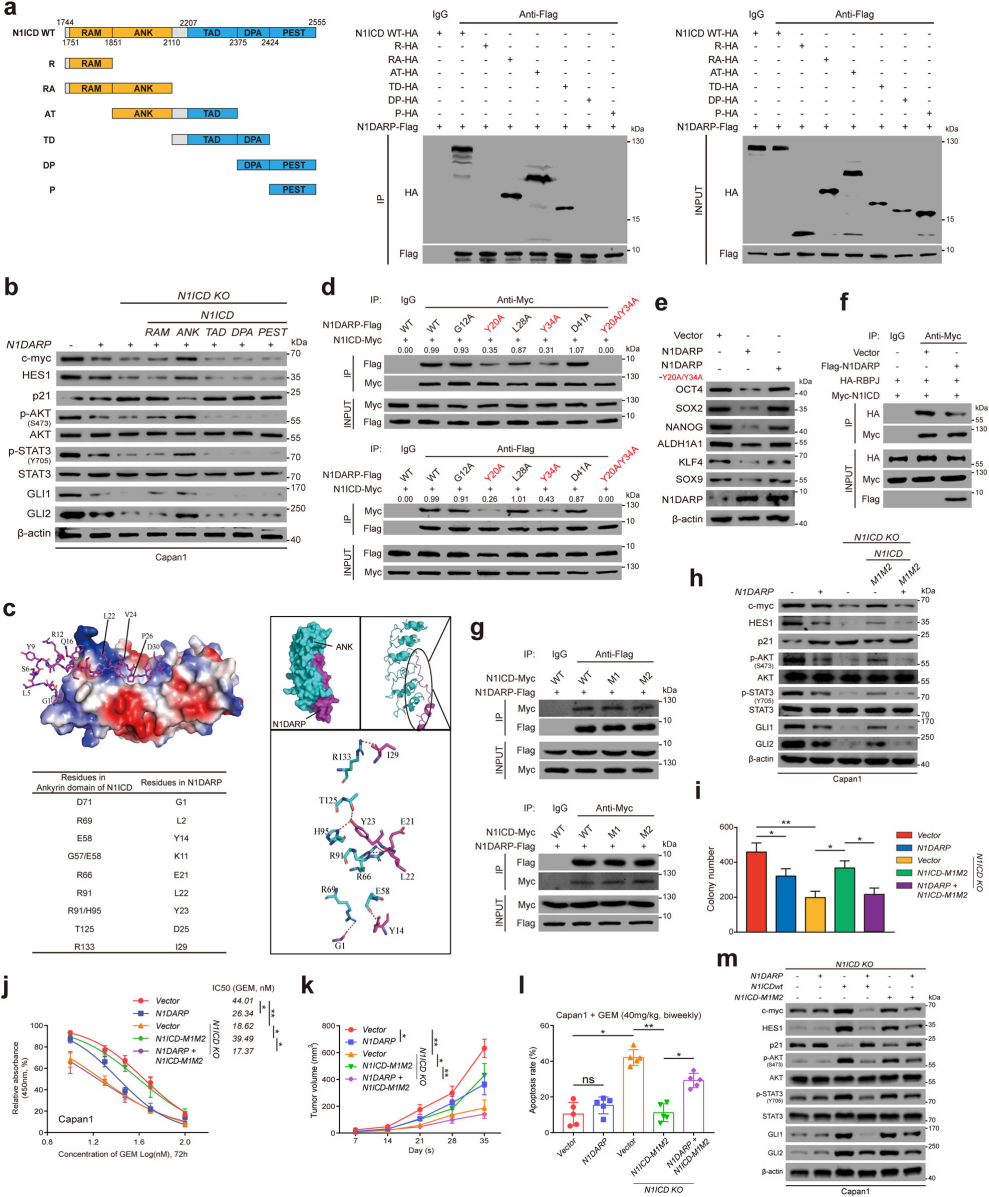
N1DARP exerts tumor suppression through interacting with and enhancing proteosome-mediated degradation of Notch1 intracellular domain. **a** Top: the strategy to construct truncated N1ICD plasmids; Bottom: co-IP followed by western blotting assay to detect N1DARP-interacting N1ICD truncations. **b** Notch signaling and its crosstalk with other pathways detected using western blotting assay using Capan1 transfected with N1DARP and individual domain of N1ICD. **c** Molecular docking using CABS-dock and PyMOL assessing electrostatic attraction and identifying key amino acids that mediate N1DARP–ANK interaction. **d** Western blotting assay detecting N1DARP–N1ICD interaction in HEK293T transfected with wild-type N1DARP or its key amino acid mutants. **e** Stem cell properties assessed by western blotting assay using Capan1 with wild-type N1DARP or its Y20/Y34 mutant. **f** The segregating effect of N1DARP on RBPJ–N1ICD interaction detected using western blotting assay. **g** N1DARP–N1ICD interaction detected using western blotting assay with Capan1 transfected with wild-type N1ICD or its M1 and M2 mutants. **h** Notch signaling and its crosstalk with other pathways detected by western blotting assay using Capan1 transfected with N1DARP or N1ICD-M1M2 mutant. **i,** **j** Cell proliferation detected using (**i**) colony formation assay and (**j**) chemosensitivity assessed using IC_50_ assay using Capan1 transfected with N1DARP or wild-type N1ICD and its M1M2 mutant. **k** The tumor volume of subcutaneously injected xenograft model using Capan1 transfected with N1DARP or N1ICD wild-type and its M1M2 mutant. **l** The apoptosis rate detected by flow cytometry using subcutaneously injected tumor derived from Capan1 transfected with N1DARP or wild-type N1ICD and its M1M2 mutant on day 35 after transplantation. **m** Notch signaling and its crosstalk with other pathways detected by western blotting assay using *N1ICD*-knockout Capan1 transfected with N1DARP and wild-type N1ICD or its M1M2 mutant. The data are presented as the mean ± SD of three independent experiments. ns no significance; *P < 0.05, **P < 0.01 by Student’s *t*-test (**i**, **l**); *P < 0.05, **P < 0.01 by one-way ANOVA (**j**, **k**).

To map the exact region mediating N1DARP and NI1ICD interactions, we constructed N1ICD mutants containing specific domains and found that only mutants with ANK or TAD domains co-precipitated with N1ICD, in which the ANK domain constituted a major proportion (Fig. 5a). Of the multiple domains of N1ICD, ANK reactivated N1DARP overexpression-induced suppression of Notch signaling and its crosstalk with other pathways, as well as cancer cell proliferation, stem cell properties, and chemosensitivity (Fig. 5b and Supplementary Fig. S5c–f), suggesting a pivotal role for the ANK domain in the N1DARP–N1ICD interaction and N1DARP-mediated tumor-suppressive function.

To further understand the N1DARP and N1ICD interactions, we performed a polarity analysis using PyMOL, which indicated that N1DARP was more prone to interact with the ANK domain, with negatively charged amino acids, such as E32, attached to positively charged regions and electrically neutral amino acids in the pocket structure of ANK (Fig. 5c). In addition, using the CABS-dock web server with flexible docking of peptides to proteins^42^, we simulated N1DARP and N1ICD interaction patterns and identified the key amino acids mediating their interaction (Fig. 5c). Next, we constructed several key amino acid mutants and found that the Y20, Y34, and Y20/Y34 double mutant obviated the N1DARP–N1ICD interaction (Fig. 5d) and the tumor-suppressive function of N1DARP (Fig. 5e and Supplementary Fig. S5g–j). Surprisingly, the interaction between N1ICD and RBPJ, a coactivator essential for canonical Notch transcriptional activity that relies on its interaction with the ANK domain, was almost competitively blocked by N1DARP (Fig. 5f). Interestingly, the functionally deficient mutants of N1ICD (M1 and M2), which abolish RBPJ-dependent transcriptional activity^43^, could still interact with N1DARP (Fig. 5g), suggesting an indispensable role for N1DARP in regulating the Notch1 pathway. Moreover, introducing the N1ICD-M1M2 mutant with N1DARP resulted in reduced tumor progression and chemosensitivity in vitro and in vivo compared with introducing the N1ICD-M1M2 mutant alone (Fig. 5h–l). This suggests that N1DARP inhibited not only the canonical but also the non-canonical Notch pathways independently of RBPJ, probably because of the robust sequestration of the ANK domain by N1DARP. Furthermore, N1DARP suppressed Notch signaling activation as well as cancer cell proliferation, stem cell properties, and chemosensitivity, mainly by interfering with the canonical Notch pathway (Fig. 5m and Supplementary Fig. S5k–n). Collectively, the above results indicate that N1DARP exerts a tumor-suppressive function by inactivating the Notch signaling pathway dependent on its interaction with and promoting the proteasome-mediated destabilization of N1ICD.

### N1DARP competitively interacts with N1ICD to impinge on USP10-mediated deubiquitination and stabilization of N1ICD

**Fig. 6:**
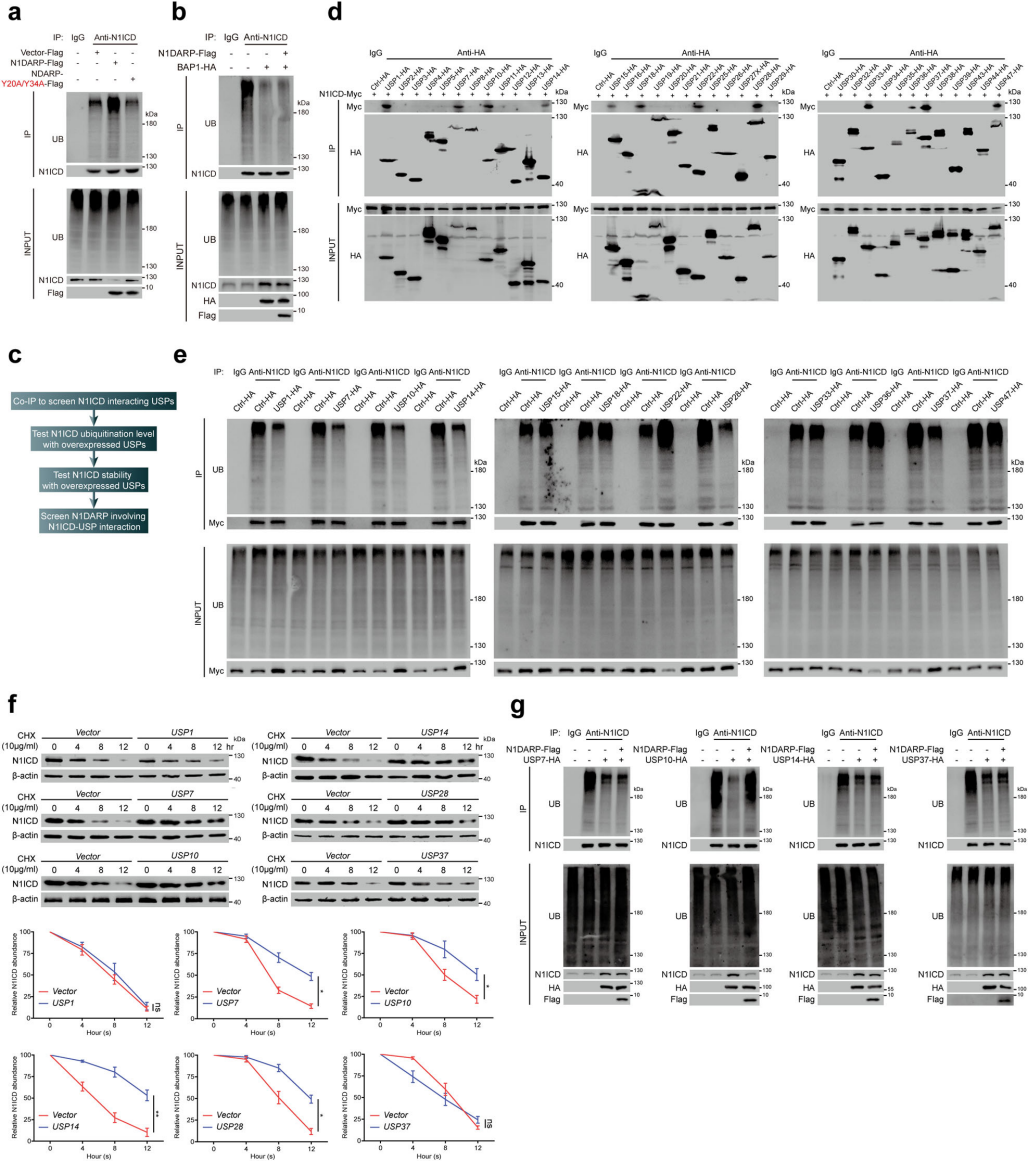
Screening of N1DARP-involving USPs-N1ICD interaction. **a** Total ubiquitination level of N1ICD detected by western blotting assay using Capan1 with wild-type or Y20/Y34 mutated N1DRAP. **b** The effect of N1DARP on the BAP1-mediated deubiquitination of N1ICD detected using western blotting assay in HEK293T. **c** Screening strategy to identify N1DARP-involved USPs-mediated deubiquitination of N1ICD. **d** Co-IP followed by western blotting assay screening N1ICD-interacting deubiquitinases in the USP family. **e** Co-IP followed by western blotting assay detecting polyubiquitination level of N1ICD using HEK293T transfected with N1ICD-interacting USPs selected above. **f** Remaining N1ICD at indicated time detected using western blotting assay in HEK293T transfected with N1ICD-interacting and deubiquitining USPs selected above after treatment with CHX. **g** The ubiquitination level of N1ICD detected using co-IP and western blotting assay in HEK293T transfected with N1ICD-interacting, deubiquitining, and destabilizing USPs (USP7, USP10, USP14, and USP37), followed by introduction of N1DARP. The data are representative of three independent experiments. ns no significance; *P < 0.05, **P < 0.01 by one-way ANOVA (**f**).

N1ICD ubiquitination plays a critical role in regulating its cleavage and stability^6^. Our results showed that the overexpression of wild-type N1DARP, but not its key amino acid mutant (N1DARP-Y20A/Y34A), promoted the polyubiquitination of N1ICD in Capan-1 cells (Fig. 6a), whereas *N1DARP* knockout inhibited the polyubiquitination of N1ICD in Panc1 cells (Supplementary Fig. S5o). More specifically, N1DARP promoted the K11- and K48-linked ubiquitination of N1ICD (Supplementary Fig. S5p), a process that converts certain proteins for proteosome-mediated degradation^44^. Therefore, we hypothesized that N1DARP destabilizes N1ICD by preventing its interaction with positive regulators, such as deubiquitinases (DUBs). The deubiquitinase BAP1 stabilizes N1ICD through the BRCA1–BARD1 complex^45^. However, we found that N1DARP was not involved in the BAP1-mediated stabilization of N1ICD (Fig. 6b). To identify the N1DARP-involving interactions, ubiquitin-specific proteases (USPs), the largest family of DUBs, were screened following a specific multi-step procedure (Fig. 6c), and N1DARP mitigated USP10-mediated deubiquitination of N1ICD (Fig. 6d–g).

**Fig. 7:**
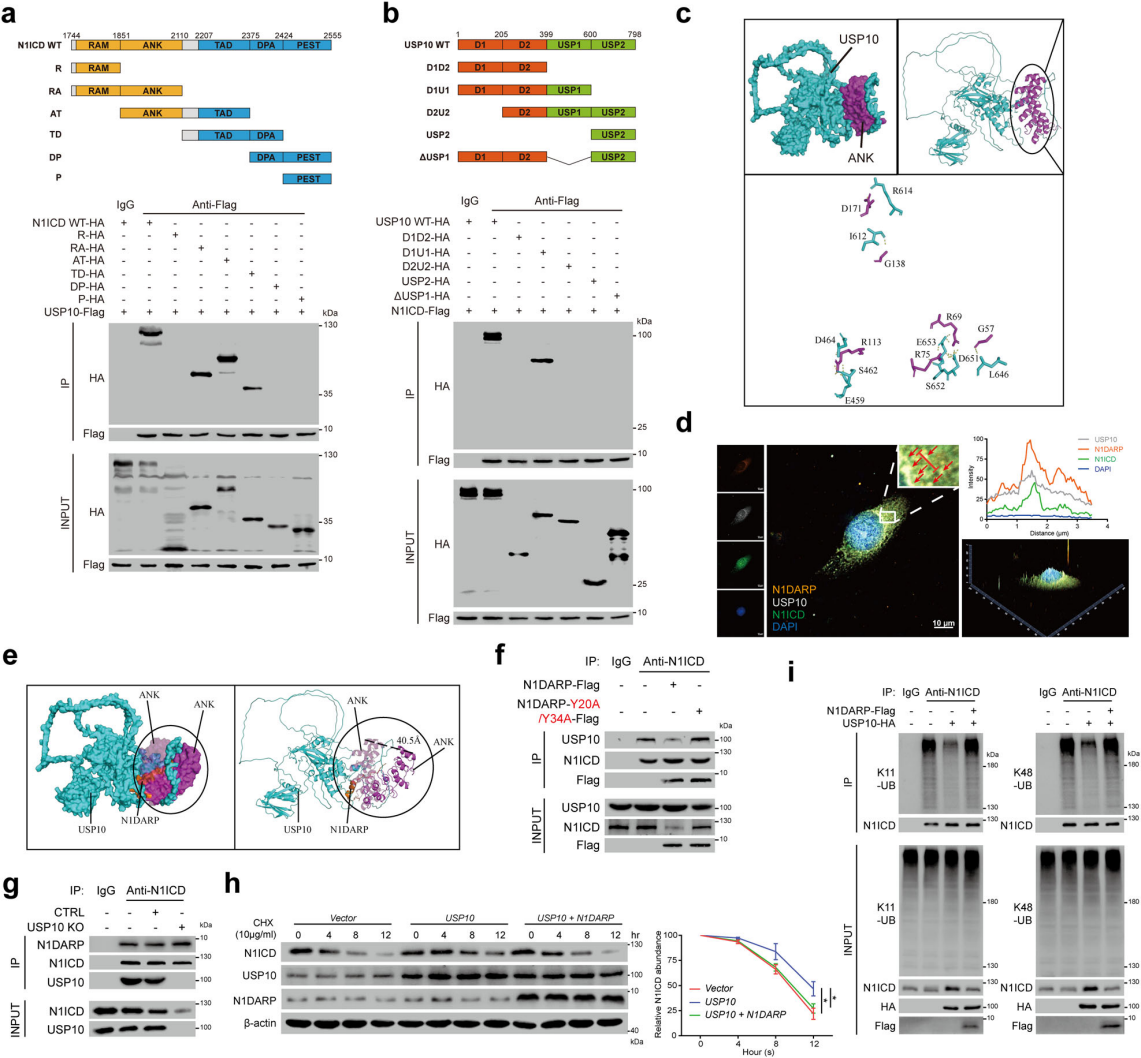
N1DARP competitively interacts with N1ICD to impinge on USP10-mediated deubiquitination and stabilization of N1ICD. **a** Top: strategy for constructing truncated N1ICD plasmids. Bottom: Co-IP followed by western blotting assay for detecting USP10-interacting N1ICD truncations. **b** Top: strategy for constructing truncated USP10 plasmids. Bottom: Co-IP followed by western blotting assay for detecting N1ICD-interacting USP10 truncations. **c** Molecular docking simulation of USP10 and ANK domain of N1ICD performed by ClusPro 2.0 and predicted key amino acids mediating USP10–ANK interaction. **d** Immunofluorescence confocal microscopy using Panc1 confirming co-localization of USP10, N1DARP, and N1ICD in the cytoplasm by detecting simultaneously enhanced fluorescence intensity of these three proteins. The red arrows indicate the co-localization site and the red line represents the fluorescence intensity measured in the right panel. **e** Molecular docking performed by ClusPro 2.0 simulating the competitive N1DARP-N1ICD binding to obstruct USP10-N1ICD interaction. **f** Co-IP followed by western blotting assay using Capan1 demonstrating that introduced wild-type N1DARP, not Y20/Y34 mutant, competed with USP10 for interaction with N1ICD. **g** K11 and K48 polyubiquitination level of N1ICD measured by western blotting assay using K11- or K48-linkage specific polyubiquitination antibody in Capan1 with USP10 overexpression followed by transfection with N1DARP. **h** Remaining N1ICD at indicated time detected by western blotting assay in Capan1 with overexpressed USP10 followed by N1DARP introduction after treatment with CHX. **i** Co-IP followed by western blotting assay using Capan1 with USP10 knockout to detect the effect of USP10 on the interaction between N1DARP and N1ICD. The data represent three independent experiments. *P < 0.05 by one-way ANOVA (**h**). Scale bars, 10 μm (**d**).

Next, to verify and elaborate on the USP10-N1ICD interaction, we performed co-IP using Capan1 and found that USP10 physically bound to N1ICD (Supplementary Fig. S6a). Immunofluorescence analysis showed that USP10 co-localized with N1ICD in the cytosol of pancreatic cancer cells (Supplementary Fig. S6b). Furthermore, in vitro pull-down and PLA assays demonstrated the direct interaction between USP10 and N1ICD (Supplementary Fig. S6c, d). Introducing wild-type USP10, but not its functionally deficient mutant (USP10-C488A)^20^, suppressed K11- and K48-linked ubiquitination and stabilized N1ICD (Supplementary Fig. S6e, f), whereas *USP10* knockout promoted K11- and K48-linked polyubiquitination of N1ICD (Supplementary Fig. S6g). Moreover, USP10 interacted with the ANK and TAD domains, with a more prominent interaction with the ANK domain. The disordered regions (D1 and D2) and the USP1 region of USP10 were both required for N1ICD binding (Fig. 7a, b). More precisely, using NetChop 3.1 to predict the cleavage sites of the proteasome in N1ICD, followed by biological validation, we found that K1945 and K2054 in N1ICD were indispensable for USP10 deubiquitination activity (Supplementary Fig. S6h, i). To specify the interactive pattern and essential interactive amino acid, we performed molecular docking using the ClusPro 2.0 web server^46^, and the diagram demonstrated the interaction of USP10 and N1ICD through electrostatic attraction, hydrogen bonding, and proper conformation (Fig. 7c and Supplementary S6j). Notably, the transcriptionally deficient N1ICD mutant (N1ICD-M1M2) did not affect its interaction with USP10 (Supplementary Fig. S6k), suggesting that USP10 regulates N1ICD deubiquitination and stability through both the canonical and non-canonical Notch pathways.

The above evidence shows that the ANK domain of N1ICD interacts with both N1DARP and USP10, and that the introduction of N1DARP abrogated USP10-mediated N1ICD deubiquitination. Therefore, we hypothesized that N1DARP inhibits USP10-mediated N1ICD deubiquitination by competitively interacting with N1ICD to prevent the USP10–N1ICD interaction. Immunofluorescence analysis performed to verify this hypothesis revealed that N1DARP, N1ICD, and USP10 were co-localized in the cytoplasm of pancreatic cancer cells (Fig. 7d). Furthermore, we performed molecular docking simulations to visualize N1ICD sequestration by N1DARP and to prevent USP10 from binding (Fig. 7e). Moreover, co-IP followed by western blotting assay showed that overexpression of wild-type N1DARP, but not the key amino acid mutant (N1DARP-Y20A/Y34A), suppressed USP10–N1ICD binding (Fig. 7f), whereas USP10 knockout had no significant effect on the N1DARP–N1ICD interaction (Fig. 7g). N1DARP consistently impeded the USP10 overexpression-induced K11- and K48-linked deubiquitination and stabilization of N1ICD (Fig. 7h, i). These findings corroborated the competitive role of N1DARP and USP10 in their interactions with N1ICD.

Next, we evaluated the role of N1DARP binding to N1ICD in USP10–N1ICD interactions during the tumorigenesis of pancreatic cancer cells. Sequencing data from the TCGA database showed an overall increase in USP10 expression in pan-cancer, especially pancreatic cancer (Supplementary Fig. S7a, b), with a 1.1% copy number amplification and gene mutations (data not shown). Moreover, upregulation of USP10 in pancreatic cancer tissues, as well as in patient serum, was observed in several GEO datasets (Supplementary Fig. S7c, d). Moreover, the diagnostic potential of USP10 was suggested using data from GSE15471, with an area under curve (AUC) of 0.873 (Supplementary Fig. S7e). Upregulated USP10 expression was associated with a worse prognosis in patients with pancreatic cancer (Supplementary Fig. S7f). Forced expression of wild-type USP10, but not its functionally deficient mutant (USP10-C488A), activated the Notch pathway and crosstalk with other pathways (Supplementary Fig. S7g). *USP10* knockout inhibited both the canonical RBPJ-dependent and non-canonical RBPJ-independent (N1ICD-M1M2) Notch pathways (Supplementary Fig. S7h). Notably, the introduction of wild-type N1DARP, but not the N1DARP-20Y/34Y mutant, ameliorated USP10 overexpression-induced Notch signaling activation, as well as tumor stemness, chemoresistance, and progression (Supplementary Fig. S7i–l). In summary, N1DARP suppresses tumor initiation, progression, and chemosensitivity by competitively impeding USP10-mediated deubiquitination and stabilization of N1ICD.

### Disrupting N1ICD-USP10 interaction by SAH-mAH2-5 attenuates progression and chemoresistance of pancreatic cancer cells

To evaluate the therapeutic potential of disrupting N1ICD–USP10 interaction by N1DARP and to define a more druggable target, we designed a stapled peptide, SAH-mAH2-5, based on the helical structure of N1DARP with a high affinity with N1ICD and remarkable physiochemical stability (Supplementary Fig. S8 and Note S1). The molecular structure of SAH-mAH2-5 is presented in Supplementary Fig. S9a. To verify the biological activity of SAH-mAH2-5, we treated Capan1 with SAH-mAH2-5 and found that SAH-mAH2-5 suppressed the deubiquitination and stability of N1ICD by counteracting the USP10–N1ICD interaction (Supplementary Fig. S9b–d). In addition, transcriptome analysis of Capan1 cells treated with SAH-mAH2-5 showed that the differentially expressed genes were mainly enriched in the Notch, Hedgehog, and Wnt/β-catenin signaling pathways (Supplementary Fig. S9e). Moreover, SAH-mAH2-5 suppressed Notch signaling and its crosstalk with other pathways in a time- and dose-dependent manner (Supplementary Fig. S9f). SAH-mAH2-5 consistently enhanced the chemosensitivity of *Capan1* and Patu8988 cells to GEM (Supplementary Fig. S9g). In vivo xenograft modeling showed retarded tumor progression with at least 2 mg/kg SAH-mAH2-5 (Supplementary Fig. S9h). Furthermore, injection of SAH-mAH2-5 increased the vulnerability of pancreatic tumor cells to GEM (Supplementary Fig. S9i).

To clarify the off-target effects of SAH-mAH2-5, we conducted a biotinylated SAH-mAH2-5 pulldown assay in PDAC-R organoids followed by Coomassie blue staining and mass spectrometry. The results showed that SAH-mAH2-5 preferentially interacted with N1ICD with limited off-target effects (Supplementary Fig. S10a). Next, we investigated whether SAH-mAH2-5 binds competitively to other Notch receptors. Surface plasmon resonance (SPR) analysis showed that the *K*_D_ value for SAH-mAH2-5 binding to N1ICD-ANK was 100–1000 times lower than that of the ANK domain of other Notch intracellular domains (N2ICD, N3ICD, and N4ICD) (Supplementary Fig. S10b). In addition, western blotting assay demonstrated that SAH-mAH2-5 did not affect the interaction of USP10 with Notch2, Notch3, or Notch4 (Supplementary Fig. S10c). Furthermore, SAH-mAH2-5 did not affect other USP10-interacting proteins (p53, KLF4, and AMPK; Supplementary Fig. S10d)^20,47,48^. Finally, to investigate the toxic effect of SAH-mAH2-5, we detected the viability of cancer and the corresponding normal cell lines from multiple Notch signaling-activated cancers and found that SAH-mAH2-5 had a general antitumor effect on Notch signaling-activated cancer cell lines from breast, lung, and colorectal cancers, but with limited effects on their corresponding normal cell lines (Supplementary Fig. S10e). Consistently, SAH-mAH2-5 suppressed Notch signaling in cancer cell lines, but not in normal cell lines (Supplementary Fig. S10f). Serum examination of mice injected with SAH-mAH2-5 revealed no abnormalities in the liver or kidney function (Supplementary Fig. S10g). Immunohistochemical analysis also demonstrated no morphological changes in normal functional organs (Supplementary Fig. S10h). Taken together, these data suggest that SAH-mAH2-5, similar to N1DARP, suppresses pancreatic cancer initiation and progression by ameliorating the Notch1 pathway, with limited off-target and toxic effects.

**Fig. 8:**
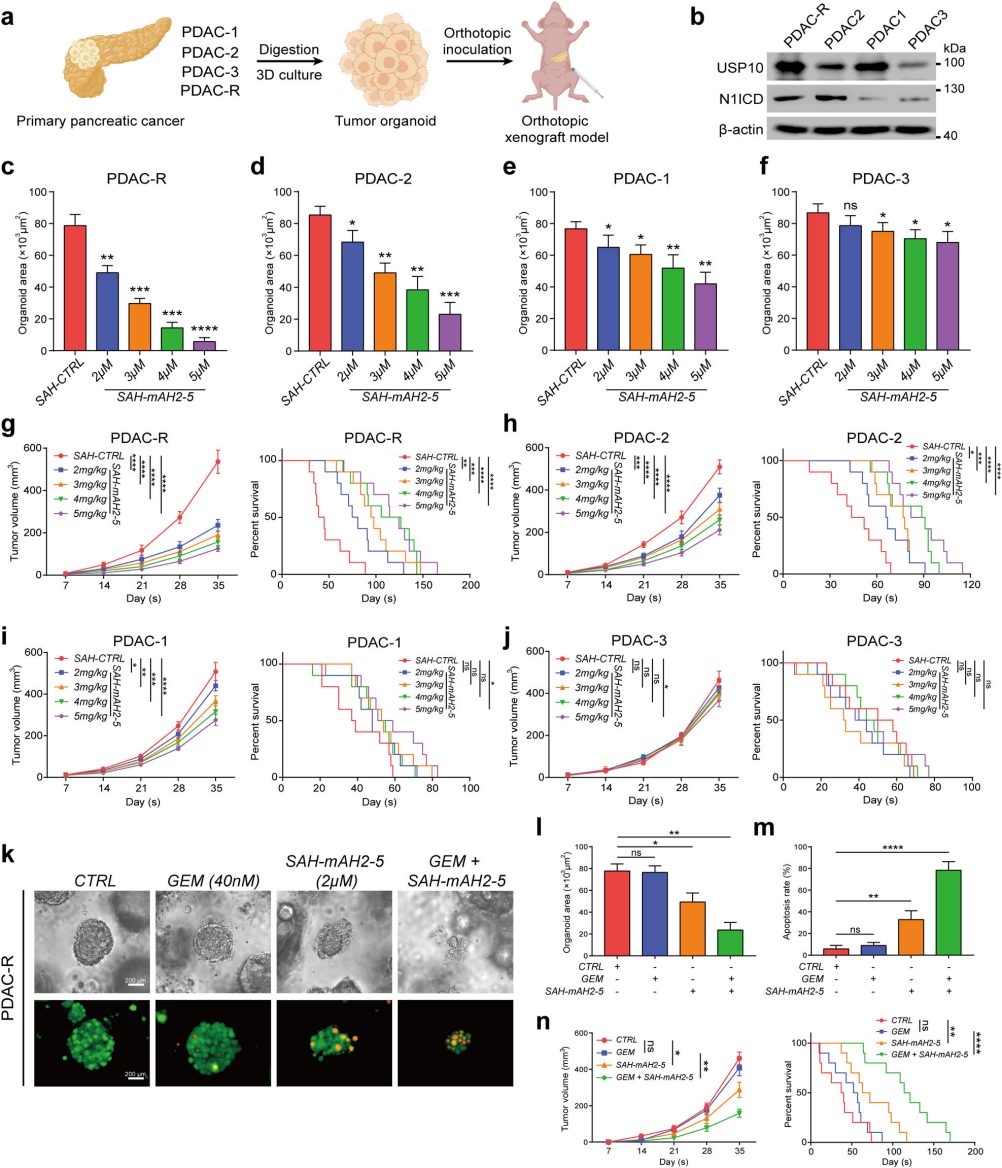
SAH-mAH2-5 suppresses progression and enhances chemosensitivity of pancreatic cancer cells in PDO and PDOX models. **a** Generation of four PDOs and orthotopically injected PDOX. **b** Protein expression levels of USP10 and N1ICD in four PDOs detected by western blot. **c**–**f** Organoid area measured by microscopy in four PDOs treated with SAH-CTRL or various concentrations of SAH-mAH2-5. **g**–**j** Orthotopic tumor volume and survival analysis of four PDOX models treated with SAH-CTRL or various concentrations of SAH-mAH2-5. **k**–**m** Organoid area and apoptosis rate stained by Calcein-AM/PI and measured using flow cytometry of GEM-resistant PDAC-R incubated with GEM, SAH-mAH2-5, or a combination of both. **n** Orthotopic tumor volume and survival analysis of PDOX model injected with PDAC-R and treated with GEM, SAH-mAH2-5, or a combination of both. The data are presented as the mean ± SD of three independent experiments. ns, no significance; *P < 0.05, **P < 0.01, ***P < 0.001, ****P < 0.0001 by Student’s *t*-test (**c**–**f**, **l**, **m**). ns no significance; *P < 0.05, **P < 0.01, ***P < 0.001, ****P < 0.0001 by one-way ANOVA (**g**–**j**, **n**). ns, no significance; **P < 0.01, ****P < 0.0001 by log-rank test (**n**). Scale bars, 200 μm (**k**).

To identify which patients with pancreatic cancer could benefit from SAH-mAH2-5 treatment, we selected and established four PDO models (PDAC-1, PDAC-2, PDAC-3, and PDAC-R) with various expression levels of USP10 and N1ICD, including one with chemoresistance to GEM (PDAC-R), for in vitro cultivation and in vivo orthotopic transplantation to establish PDOX models (Fig. 8a, b). SAH-mAH2-5 treatment inhibited organoid growth in a dose-dependent manner (Fig. 8c–f). Notably, PDAC-R and PDAC-2 with higher or moderate USP10 and N1ICD levels showed substantial suppression of organoid growth, whereas the effect on PDAC-1 with higher USP10 but lower N1ICD levels was less pronounced, and almost no effect was evident on PDAC-1, which showed the lowest levels of USP10 and N1ICD (Fig. 8c–f). Consistently, the injection of SAH-mAH2-5 into the four orthotopic PDOX models retarded tumor progression and prolonged the median survival time of the mice. PDAC-R exhibited the most significant antitumor effect, with PDAC-2 and PDAC-1 exhibiting moderate effects, whereas PDAC-3 showed almost no response (Fig. 8g–j). In addition, combined treatment with GEM and SAH-mAH2-5 alleviated chemoresistance and induced apoptosis in PDAC-R (Fig. 8k–m). Moreover, pre-injection of SAH-mAH2-5 sensitized PDAC-R cells to GEM and prolonged the median survival of tumor-bearing mice (Fig. 8n). Collectively, these results indicate that patients with pancreatic cancer with high USP10 or N1ICD protein levels could potentially benefit from SAH-mAH2-5 treatment.

## Discussion

Although target-based therapy has progressed in the past decades, promising therapeutic targets derived from known protein databases seem to be exhausted^29^; therefore, it is essential to explore new targetable molecules for cancer treatment. Recently, microproteins or polypeptides produced by non-coding RNAs have provided a novel resource bank for targeted therapeutic options for cancer patients. Various polypeptides derived from non-coding RNAs have been reported to regulate tumorigenesis and cancer development^32–34^. However, few non-coding RNA-based therapeutic options for pancreatic cancer have been explored. Our study identified *LINC00261*, a newly emerging pan-cancer suppressor^49^, as a functionally translatable lncRNA based on evidence from combined analyses of mRNA and ribosome sequencing using patient-derived organoid models. In preliminary screening, we focused on an evolutionarily conserved peptide encoded by *LINC00261*, N1DARP, comprising 41 amino acids, which is consistent with previous evidence that polypeptides with more than 30 amino acids are considered potentially functional^38,39^. Overexpression of N1DARP suppresses the initiation and progression of pancreatic cancer and increases the vulnerability of cancer cells to GEM, confirming the tumor-suppressive role of N1DARP. Deprivation of N1DARP from *LINC00261* partially rescued tumor growth and entirely rescued stemness traits of pancreatic cancer cells mediated by *LINC00261* overexpression, suggesting that *LINC00261* could partially regulate tumor growth, while *LINC00261* encodes N1DARP as a peptide that plays a major role in controlling cell proliferation and entirely mediates stemness traits of pancreatic cancer. Nevertheless, further robust evidence based on transgenic mice is required to fully elaborate on this issue. In addition, in contrast to previously reported polypeptides that are mostly oncogenic^32–34^, N1DARP is a tumor-suppressive microprotein that may provide a novel approach for protein delivery-based cancer therapy.

Mechanistically, we found that N1DARP suppressed canonical Notch1 signaling by interacting with and promoting the proteasome-mediated degradation of N1ICD, providing a novel regulator of Notch1 signaling. The pharmacological inhibition of Notch signaling is a promising therapeutic approach for pancreatic cancer. Recently, γ-secretase inhibitors aimed at blocking Notch receptor cleavage, including PF-03084014, MK-0752, and RO4929097, have been administered in clinical studies to treat locally advanced or metastatic pancreatic cancer; however, no significant remission has been observed^13–15^. Dual- or pan-Notch inhibition by γ-secretase inhibitors, which inhibit Notch1 and other family members, may cause profound compensatory activation of other signaling pathways, leading to chemoresistance or adverse effects. These findings emphasize the need for a practical approach specifically targeting individual Notch receptors. Recently, a phase II study showed that tarextumab, a Notch2/3 inhibitor, markedly reduced pancreatic cancer when combined with GEM and paclitaxel^18^. Similarly, our study provides a novel perspective for targeting specific Notch1 receptors using polypeptides encoded by non-coding RNAs. Notch1 undergoes rapid degradation, mainly through the ubiquitin-proteasome system, and a variety of factors regulate Notch1 stability^17^. Therefore, we hypothesized that small molecules that promote the degradation of Notch1 or interfere with its transcriptional complex could be a promising alternative strategy to target Notch1-activated cancers. The present study confirmed that the microprotein N1DARP promotes N1ICD degradation by increasing K11- and K48-linked polyubiquitination of N1ICD, suggesting that N1DARP could be a novel therapeutic target for pancreatic cancer that specifically deactivates the Notch1 pathway. Furthermore, N1DARP competitively interacted with N1ICD to disrupt the USP10–N1ICD interaction, thus promoting the proteasome-mediated degradation of N1ICD. USP10–N1ICD interactions play a vital role in multiple biological processes other than cancer-related chemoresistance, such as cell development, angiogenesis, and cardiac dysfunction^26–28^. Therefore, targeting this interaction could be a novel approach for eradicating pancreatic cancer. To define a more druggable target at the interface between USP10 and N1ICD, we generated a stapled peptide, SAH-mAH2-5, derived from one of the α-helices of N1DARP, with remarkable affinity, permeability, and physiochemical stability. SAH-mAH2-5 preferentially interacts with N1ICD and disrupts the USP10–N1ICD interaction. Treatment with SAH-mAH2-5, which had a synergistic effect with GEM, suppressed tumor growth and extended the survival of mice, especially those with higher USP10 and N1ICD expression. These findings provide strong evidence for the therapeutic potential of targeting the USP10–N1ICD interaction, offering a promising chemical structure and combination strategy for the treatment of pancreatic cancer.

Despite these interesting findings, this study had several limitations. First, although we performed several experiments to prove that N1DARP is a critical tumor suppressor independent of *LINC00261*, which encodes N1DARP, these experiments were superficial and not decisive, as shown in Fig. 2 and Supplementary Fig. S4. A genetically engineered mouse model with a mutated start codon for N1DARP is required to confirm these results. Second, the designed stapled peptide, SAH-mAH2-5, exhibited profound anti-pancreatic cancer effects, especially in Notch1-hyperactivated pancreatic cancer, in multiple preclinical models. However, clinical trials are required to examine the effective concentration, therapeutic window, and systematic toxicity of SAH-mAH2-5 in humans to justify its translational potential. In addition, as shown in Supplementary Fig. S10e, f, some in vitro tests preliminarily demonstrated that SAH-mAH2-5 is a remarkable antitumor agent for breast, lung, and colon cancers other than pancreatic cancer. Nevertheless, whether SAH-mAH2-5 can present identical or similar antitumor effects in vivo or whether it is also effective for hematological malignancies that are mostly caused by Notch1 gain-of-function mutations needs further validation.

Collectively, the results of this study identified N1DARP as a novel tumor suppressor and chemotherapy sensitizer for use in pancreatic cancer therapy by controlling USP10-Notch1 oncogenic signaling and provided insights into a novel strategy that targets USP10–Notch1 interaction by a designed stapled peptide SAH-mAH2-5 derived from the microprotein N1DARP (Supplementary Fig. S11). Targeting the N1DARP–N1ICD interaction and N1ICD degradation could be a promising alternative strategy for treating Notch1-activated pancreatic cancer and may have broader applications in the treatment of non-cancerous diseases.

## Materials and methods

### Patient samples

Seventy-five pairs of pancreatic cancer tissues and corresponding adjacent normal tissues were harvested from patients at Ruijin Hospital Affiliated with Shanghai Jiaotong University School of Medicine. All enrolled patients met the following criteria: (i) pathological diagnosis of pancreatic adenocarcinoma, (ii) relatively complete clinicopathological and follow-up data, and (iii) no preoperative chemotherapy. Written informed consent was obtained from all patients involved, and the study protocol was approved by the Ethics Committee of Ruijin Hospital (No.161 in 2021).

### Establishment of patient-derived pancreatic cancer organoids

Following the approved procedure mentioned above, the normal pancreatic tissue was resected, and the remaining pancreatic cancer tissue was minced and subjected to enzymatic digestion with collagenase (1 to 2 mg/mL; Sigma-Aldrich St Louis, MO, USA, C9407) on an orbital shaker at 37 °C for 1 to 2 h. The suspension was strained through a 100-μm filter to retain the tissue fragments. The organoid fraction was obtained after centrifugation at 530× *g* for 5 min, resuspension in RPMI 1640 medium mixed with cold Matrigel (BD Biocoat, 356234, 10 mg/mL), and solidification in pre-warmed 24-well suspension culture plates at 37 °C for 20 min. Following complete gelation, 400 mL of BC organoid medium was added to each well, and the plates were transferred to humidified 37 °C/5% CO_2_ incubators containing either 2% or ambient O_2_. The medium was changed every four days, and the organoids were passaged every one to four weeks.

### Cell culture

Pancreatic cancer cell lines (Aspc1, Bxpc3, Capan1, Mia-PaCa2, Panc1, Sw1990, and Patu8988) and the h-TERT transfected immortalized human pancreatic ductal epithelial cell line (HPNE), breast cancer and the corresponding normal cell line (MDA-MB-231 and MCF-10A), lung cancer and its normal cell line (A549 and BEAS-2B), and colorectal cancer and its normal cell line (HCT116 and NCM460) were purchased from the Cell Bank of the Chinese Academy of Sciences. The cells were authenticated by STR, tested negative for mycoplasma, and maintained in RPMI 1640, DMEM, and IMDM supplemented with 10% fetal bovine serum and antibiotics.

### Generation of N1DARP^loxP/loxP^; LSL-Kras^G12D/+^; LSL-Trp53^R172H/+^ mice

*N1DARP* knockout mice were generated by Cyagen Biosciences, Inc. (Shanghai, China). The mouse genomic DNA sequence of the *N1DARP* gene was verified using bioinformatics analysis. Based on the genomic DNA sequence, strategies for gene targeting and construction of a Cas9/sgRNA plasmid were established to delete *N1DARP*. Embryonic stem cells (ESCs) were electroporated with the *N1DARP* knockout vector, and transformants were identified by PCR and Southern blot analysis. The selected ESCs were injected into C57BL/6 blastocysts, and the embryos were transferred into the pseudopregnant uterus of a C57BL/6 mouse to obtain a chimeric mouse. PCR was performed to identify heterozygous offspring of the C57BL/6 mice. *N1DARP* knockout mice were then crossed with congenic LSL-Kras^G12D/+^; LSL-Trp53^R172H/+^; Pdx1-Cre mice, a widely investigated spontaneous model of pancreatic cancer, to generate LSL-Kras^G12D/+^; LSL-Trp53^R172H/+^; N1DARP^loxP/loxP^; Pdx1-Cre mice.

### Peptide synthesis

All peptides were manufactured by Chinese Peptide, Inc. (Hangzhou, China). The synthetic peptides were purified to > 98% purity by high-pressure liquid chromatography for both in vitro and in vivo applications. Stapled SAH-mAH2-5 peptides were formed by incorporating mAH2-5 peptide with two units of non-natural alkenyl amino acids S5 at the relative positions *i* and *i* + 7, and then cross-linked by ring-closing olefin metathesis, resulting in a stapled peptide that is braced in a α-helical conformation. For in vitro experiments, the peptides were dissolved in phosphate buffered saline to generate a 4 mM stock solution. For in vivo use, samples were dissolved in PBS and stored on ice until injection. Before injection, the solution was brought to room temperature at 25 °C.

### SPR analysis

The binding kinetics between N1DARP or SAH-mAH2-5 and the indicated peptides were analyzed using a SPR assay with a BIAcore T200 instrument (GE Healthcare, Pittsburgh, PA, USA). The dissociation constant was calculated using BIA-evaluation software.

### Bioinformatic analysis

GSEA was performed to reveal the enriched hallmarks or pathways in Capan1 cells transfected with empty vector or N1DARP, and Capan1 cells treated with SAH-CTRL or SAH-mAH2-5. USP10 expression was analyzed using GEO datasets GSE16515, GSE15471, GSE71729, GSE62452, GSE1542, and GSE101448. Differential analysis was performed using the GEO2R online tool. The expression and prognostic data of USP10 were also gathered and analyzed using Gene Expression Profiling Interactive Analysis, which is based on the Cancer Genome Atlas and Genotype-Tissue Expression project.

### Statistical analysis

Statistical details are indicated in the figure legends, text, and this section. Data were analyzed using Fisher’s exact test, Student’s *t*-test (or paired Student’s *t*-test), one-way ANOVA with Tukey’s test, Kruskal–Wallis test, or Mann–Whitney *U* test, as indicated. The results are presented as the mean ± standard error of the mean (SEM) or standard deviation (SD). Error bars indicate SD or SEM of at least three independent experiments. Survival analysis was performed using the Kaplan–Meier method and the log-rank test. *P* < 0.05 was considered statistical significance. All statistical analyses were performed using SPSS 23.0 and GraphPad Prism 7.0.

### Supplementary information

Supplementary information

## Data Availability

The data that support the findings of this study are available from the corresponding author upon reasonable request. The datasets used and/or analyzed during the current study are available at the NCBI Sequence Read Archive, under BioProject PRJNA851223. Additional materials and methods are described in the Supplementary Data S1.
