# Supplementary material for: A microprotein N1DARP encoded by LINC00261 promotes Notch1 intracellular domain (N1ICD) degradation via disrupting USP10-N1ICD interaction to inhibit chemoresistance in Notch1-hyperactivated pancreatic cancer
Source: Cell Discov. 2023 Sep 15;9:95. doi: 10.1038/s41421-023-00592-6 (PMC10504324; doi:10.1038/s41421-023-00592-6)
Supplement: Supplementary file 1 — Supplementary information [file 41421_2023_592_MOESM1_ESM.pdf]

Supplementary Materials for

N1DARP promotes N1ICD degradation via disrupting USP10-N1ICD interaction to inhibit chemoresistance in Notch1-hyperactivated pancreatic cancer

Shuyu Zhai, Jiewei Lin, Yuchen Ji, Ronghao Zhang, Zehui Zhang, Yizhi Cao, Yang Liu, Xiaomei Tang, Jia Liu, Pengyi Liu, Jiayu Lin, Fanlu Li, Hongzhe Li, Yusheng Shi, Da Fu, Xiaxing Deng, Baiyong Shen

Correspondence to: Baiyong Shen. shenby@shsmu.edu.cn

Correspondence to: Xiaxing Deng. dxx10716@rjh.com.cn

Correspondence to: Da Fu. fuda@shsmu.edu.cn

Correspondence to: Yusheng Shi. 815212808@qq.com

This PDF file includes:

Supplementary Data S1. Materials and methods

Supplementary Note 1. Generation and modification of N1DARP-derived stapled peptide SAH-mAH2-5.

Supplementary Figure S1. Profiling of expression and translation of lncRNAs in pancreatic cancer vs. normal pancreatic cells.

Supplementary Figure S2. N1DARP is endogenously expressed in pancreatic cancer cells.

Supplementary Figure S3. N1DARP expression is inhibited in pancreatic cancer cells and denotes better prognosis.

Supplementary Figure S4. N1DARP is essential for LINC00261 tumor suppressive function.

Supplementary Figure S5. ANK repeat sequence serves as a pivotal domain for N1DARP tumor suppressive function.

Supplementary Figure S6. USP10 interacts with and promotes deubiquitination of N1ICD.

Supplementary Figure S7. USP10 upregulated in pancreatic cancer promotes tumor progression and chemoresistance by deubiquitinating and stabilizing N1ICD.

Supplementary Figure S8. Generation and modification of N1DARP-derived stapled peptide SAH-mAH2-5.

Supplementary Figure S9. SAH-mAH2-5 suppressed tumor initiation and progression by disrupting USP10-N1ICD interaction.

Supplementary Figure S10. Off-target and toxic effects of SAH-mAH2-5.

Supplementary Figure S11. Graphical summary

Supplementary Table S1. The correlation between N1DARP expression and clinicopathological features of patients with pancreatic cancer.

Supplementary References

Supplementary Data S1

Materials and methods

Antibody generation and western blotting

A polyclonal antibody against the 41-aa peptide N1DARP, encoded by *LINC00261* was obtained by inoculating the peptides into rabbits. The antibody was purified by affinity chromatography. Cell samples were lysed with RIPA buffer mixed with protease and phosphatase inhibitor cocktails. The proteins were separated by 10% SDS-PAGE and transferred onto PVDF membranes. Images were generated using a Tanon 5200 chemiluminescence imaging system (Tanon, Shanghai, China) and quantified by densitometry using the ImageJ software. Primary and appropriate secondary antibodies were used to detect the corresponding proteins. Primary antibodies used were anti-N1DARP (newly generated; 1:500 for WB, 1:50 for IF and 1:100 for IP), anti-GFP (CST, #2037, 1:1000), anti-Flag (Proteintech, 80010-1-RR, 1:1000), anti-HA (Proteintech, 51064-2-AP, 1:1000), anti-MYC (Proteintech, 16286-1-AP, 1:1000), anti-USP10 (CST, #8501, 1:1000 for WB, 1:200 for IF, and 1:200 for IP), anti-β-actin (CST, #3700, 1:1000), anti-Ki-67 (CST, #9449, 1:200 for IHC), anti-SOX2 (CST, #14962, 1:200 for IHC and1:1000 for WB), anti-OCT4 (CST, #2890, 1:1000), anti-NANOG (CST, #4903, 1:1000), anti-ALDH1A1 (CST, #54135, 1:1000), anti-KLF4 (Proteintech, 11880-1-AP, 1:1000), anti-SOX9 (CST, #82630, 1:1000), anti-N1ICD (CST, #4147, 1:1000 for WB, 1:100 for IF and 1:200 for IP), anti-c-myc (CST, #13987, 1:1000), anti-HES1 (CST, #11988, 1:1000), anti-p21 (CST, #2947, 1:1000), anti-p-AKT (CST, #4060, 1:1000), anti-AKT (CST, #4691, 1:1000), anti-p-STAT3 (CST, #9145, 1:1000), anti-STAT3 (CST, #30835, 1:1000), anti-GLI1 (CST, #3538, 1:1000), anti-GLI2 (CST, #66670, 1:1000), anti-total polyubiquitin (CST, #58395, 1:2000), anti-K6 polyubiquitin (ABclonal, A18106, 1:1000), anti-K11 polyubiquitin (ABclonal, A18197, 1:1000), anti-K27 polyubiquitin (Abcam,

ab181537, 1:1000), anti-K29 polyubiquitin (ABclonal, A18198, 1:1000), anti-K33 polyubiquitin (ABclonal, A18199, 1:1000), anti-K48 polyubiquitin (Abcam, ab140601, 1:1000), and anti-K63 polyubiquitin (Abcam, ab179434, 1:1000). Secondary antibodies used were anti-rabbit IgG (Proteintech, SA00001-2, 1:5000) and anti-mouse IgG (Proteintech, SA00001-1, 1:5000).

Plasmid construction and transient cell transfection

Oligonucleotide synthesis and subcloning have been described previously [1]. Briefly, Genomic DNA was extracted from pancreatic cancer tissue as a template. The open reading frame of N1DARP was amplified by RT-PCR using the following primers: (Forward) 5′-GTAGGACTCAAGTGGATTGTCCTGGCAGG-3′ and (Reverse) 5′-TGAAGTCAGACTCGCAGGCGTGCATCGCAC-3. ’ pGEM-N1DARP was generated by subcloning the 140bp N1DARP PCR product into the pGEM-T-Easy vector. The start codon-mutated N1DARP was derived from pGEM-N1DARP using the QuickChange method. The following primers were used to amplify the mutated N1DARP: (Forward) 5′-TTCACTGGAGAGGGGTCGCTCAGTGTATCAGG-3′ and (Reverse) 5′-CACCTCTGGAGCTATCAGACCCTGTCCAGCAA-3. ’ pcDNA3.1-N1DARP and pcDNA3.1-N1DARPmut were synthesized by subcloning N1DARP and N1DARP with start codon mutations, respectively, into the HindIII and KpnI sites of pcDNA3.1 eukaryotic expression vector. To generate a plasmid construct fused with the wild-type or start codon mutant (ATG to ATT) GFP (GFPmut) and FLAG, full-length complementary cDNAs of ORF12 and the start codon mutant (ATG to ATT) of ORF12 (ORF12mut) were synthesized and subcloned into the expression vector pcDNA3.1-GFP. In this vector, the GFP start codon ATG was mutated to ATT (Bioegene, Shanghai, China) to produce pcDNA3.1-ORF12-GFPmut and pcDNA3.1-ORF12mut-GFPmut. Full-length complementary

cDNAs of ORF12 and the start codon mutant (ATG to ATT) of ORF12 were synthesized and subcloned into the expression vector pcDNA3.1-Flag (Bioegene) to generate pcDNA3.1-ORF12-Flag and pcDNA3.1-ORF12mut-Flag, respectively. In addition, key amino acid mutants mediating the N1DARP-ANK interaction (G12A, Y20A, L28A, Y34A, D41A, and Y20A/Y34A) were cloned into the pcDNA3.1-Flag vector by standard subcloning. Full-length N1ICD-HA and truncations of N1ICD, including R-HA (amino acids 1744–1851), RA-HA (amino acids 1744–2110), AT-HA (amino acids 1851–2375), TD-HA (amino acids 2110–2424), DP-HA (amino acids 2375–2555), and P-HA (amino acids 2424–2555), were constructed into pcDNA 3.1-HA vector by standard subcloning. N1ICD-M1 and-M2 mutants were constructed as described previously [2]. Full-length USP10-HA and truncated USP10, including D1D2-HA (amino acids 1–399), D1U1-HA (amino acids 1–600), D2U2-HA (amino acids 205–798), USP2-HA (amino acids 600–798), and ΔUSP1-HA (amino acids 1–399 and 600–798), were cloned into the pcDNA 3.1-HA vector by standard subcloning. USP10-C488A-HA was cloned into pcDNA 3.1-HA. The Lys site mutants of N1ICD interacted with USP10 as predicted by NetChop (K1780A, K1781A, K1782A, K1798A, K1945A, K2049A, K2054A, K1821A, K1822A, K2156A, K2157A, K2160A, K2164A, K2171A, K2177A, K2181A, K2182A, and K2187A) using a Fast Mutagenesis System (TransGen Biotech, Beijing, China). Deubiquitinase BAP1 and USP family members USP1–USP47 tagged with HA were purchased from Sino Biological, Inc. (Beijing, China). The Halo-GFP-Mito plasmid was designed and constructed as previously described. Briefly, the C-terminus of Haloenzyme–GFP was fused with the mitochondrial outer membrane-targeting domain comprising the C-terminal 47 amino acids of the *Listeria monocytogenes ActA* gene, which anchors Halo-GFP to the outer mitochondrial membrane and is oriented in the cytosol. Transient transfections of HET293T or pancreatic cancer cell lines was performed using Lipofectamine 3000 (Thermo Fisher

Scientific, Waltham, MA, USA) according to the manufacturer's protocol. Twelve hours before transfection, cells were seeded in 12-well plates at a density of 2 × 105 cells/well. Cells were transfected when they reached 90%-95%. The cells were transfected with 4 μL/well of corresponding pcDNA3.1 vector with 2 μL Lipofectamine 3000.

CRISPR-Cas9 mediated gene knockout

An online tool (http://crispr.mit.edu/) was used to design the target sequences of guide RNA (gRNA). gRNAs targeted exon 4 of *LINC00261* and full-length N1DARP. The highest-scoring designs were synthesized using in silico prediction and screening. The sequences were as follows: gLINC00261-1: 5'-GAGGCTTGACACAGGTATATATGAC; gLINC00261-2: 5'-TTCAAGACACTCTAGGCGCCTAATC; gN1DARP-1: 5'- AGAAGACCTTCATAAGGGCAGGG; gN1DARP-2: 5'- GCCCACTTCAGAGTCGAGTCAGG. To produce the CRISPR lentivirus, 10 μg lenti-CRISPR (v2) gRNA or lenti-CRISPR (v2) CTRL along with Lipofectamine 3000 (Thermo Fisher Scientific) were added to HEK293RT cells seeded in 100-mm plates. After incubation for 48–72 h, the supernatants containing lentiviruses were collected and used to transfect Panc1 cells expressing the Cas9 protein. Transfected cells were selected using blasticidin for Cas9 expression and subjected to flow cytometry to pool the EGFP-positive population with the corresponding gRNA.

Establishment of stably expressed cell lines and organoids

Lentiviral vectors expressing wild-type *LINC00261*, *LINC00261* with start codon mutant (ATG to ATT) of N1DARP (LINC00261-N1DARPmut), wild-type N1DARP, and start codon mutant (ATG to ATT) of N1DARP (N1DARPmut) were transfected into HEK293T cells together with packaging vectors

psPAX2 (Addgene, Watertown, MA, USA) and pMD2G (Addgene) for lentivirus generation through Lipofectamine 3000 (Thermo Fisher Scientific) according to the manufacturer's instructions. Wild-type Capan-1 or Capan-1 with *LINC00261* knockout were transfected with the corresponding lentiviruses mentioned above, along with polybrene (5 mg/ml). After incubation for 24 h, the cells were selected using 10 mg/ml blasticidin for 5 days. Similarly, to establish stably transfected tumor organoids, 3D cultured patient-derived organoids (PDAC-1 and PDAC-2) were digested into single cells or fragments and plated in a 2D culture environment. After adherence, the cells were transfected with the aforementioned lentiviruses, along with polybrene (5 mg/ml), and selected using 10 mg/ml blasticidin for 5 days. The remaining organoids were digested again and maintained in Matrigel until further passaging. This process is illustrated in Fig 2a.

Quantitative real-time PCR (qRT-PCR)

Total RNA from pancreatic tissues and cell lines was extracted using the TRIzol Reagent (Invitrogen, Carlsbad, CA, USA). RNA from nuclear and cytoplasmic fractions was separated using a PARIS Kit. Reverse transcription was performed using HiScript III RT SuperMix (Vazyme, Nanjing, China). The AceQ Universal SYBR qPCR Master Mix (Vazyme) was used to perform qRT-PCR. The expression of β-actin was used as an internal mRNA control. Primers are listed as follows: Notch1 forward, 5'-TGGACCAGATTGGGGAGTTC-3'; Notch1 reverse, 5'- GCACACTCGTCTGTGTTGAC-3'; Notch2 forward, 5'-CAACCGCAATGGAGGCTATG-3'; Notch2 reverse, 5'-GCGAAGGCACAATCATCAATGTT-3'; Notch3 forward, 5'-CGTGGCTACACTGGACCTC-3'; Notch3 reverse, 5'-AGATACAGGTGAACTGGCCTAT-3'; Notch4 forward, 5'-CCTGGCTCCTTCAACTGCC-3'; Notch4 reverse, 5'-GCAAGTAGGTCCAGACAGGT-3'; MYC

forward, 5’-TCCCTCCACTCGGAAGGAC-3’; MYC reverse, 5’-CTGGTGCATTTTCGGTTGTTG-3’; HES1 forward, 5’-CCTGTCATCCCCGTCTACAC-3’; HES1 reverse, 5’-CACATGGAGTCCGCCGTAA-3’; CDKN1A forward, 5’-TGTCCGTCAGAACCCATGC-3’; CDKN1A/p21 reverse, 5’-AAAGTCGAAGTTCCATCGCTC-3’.

Cell proliferation assay

Equal numbers of stably transfected pancreatic cancer cells were plated in 96-well plates (2000 cells/well). Cell viability was measured using a CCK8 (Dojindo, Kumamoto, Japan). A colony formation assay (1000 cells/well in 6-well plates) was performed to determine the proliferative capacity of pancreatic cancer cells. In addition, a Cell-Light EdU DNA cell proliferation kit (RiboBio, Guangzhou, China) was used to detect cell proliferation potential, following the manufacturer’s instructions.

Tumor sphere formation assay

Stably transfected pancreatic cancer cells (Capan1 or Panc1) were digested and gently resuspended in a single-cell suspension of StemXVivo Serum-Free Tumorsphere Media (R&D Systems, Minneapolis, MN, USA) containing heparin and hydrocortisone. Ten thousand cells were resuspended in 1 ml complete StemXVivo medium and transferred to each well of an ultra-low adhesion 12-well plate. The cell culture plate was then moved into a 37°C cell culture incubator and maintained for 7 days.

Immunofluorescence (IF) and colocalization assay

For pancreatic cancer cell lines, Capan1 and Panc1 seeded on coverslips were briefly washed with PBS

and fixed with 4% buffered paraformaldehyde for 15 min, permeabilized with 0.5% Triton X100 for 15 min, blocked with 3% BSA for 30 min at 25℃, and then stained with N1DARP, USP10, and N1ICD antibodies followed by corresponding secondary antibodies. Nuclei were counterstained with DAPI. To prepare pancreatic cancer organoids, PDAC-1 cells cultured in Matrigel were briefly washed with PBS, dispersed, and fixed in 4% buffered paraformaldehyde. The cells were then embedded in agarose, fixed, and sliced. The sections were blocked with 3% BSA and stained with N1DARP, USP10, and N1ICD antibodies, followed by incubation with the corresponding secondary antibodies. Nuclei were counterstained with DAPI. The images were captured using a confocal fluorescence microscope (Olympus Microsystems, Tokyo, Japan). Quantitative image analysis was performed using ZEN 2.0 software.

Immunohistochemistry (IHC) analysis and tissue microarray

Pancreatic tumor tissue sections were analyzed by two pathologists who were blinded to the patient's identity. The sections were deparaffinized with xylene, rehydrated in an ethanol series, submerged in EDTA antigenic retrieval buffer (pH 8), and microwaved for antigenic retrieval. Subsequently, the sections were treated with 3% hydrogen peroxide in methanol to quench endogenous peroxidase activity, followed by incubation with 1% goat serum albumin to block non-specific binding. Tissue sections were incubated with the corresponding antibodies overnight at 4°C. After washing, the tissue sections were treated with goat anti–mouse/rabbit IgG horseradish peroxidase polymer for 20 min. 3, 3'-Diaminobenzidine was used as the chromogen. Tissue microarrays of seventy-five paraffin-embedded pancreatic cancer tissue sections were prepared according to manufacturer's instructions. Briefly, tissue cylinders (diameter, 0.6 mm) were obtained from selected regions of the donor block and punched precisely into a recipient paraffin block using a tissue arraying instrument (Beecher

Instruments, Sun Prairie, WI, USA). Consecutive 5 μm sections of the microarray blocks were made with a microtome. The H-scores were determined by combining the intensity of staining with the proportion of positively stained tumor cells. The intensity was graded as follows: 0 = negative; 1 = weak; 2 = moderate; and 3 = strong. The proportion of positive tumor cells was graded: 0, 0%–5%; 1, 5%–25%; 2, 26%–50%; 3, 51%–75%; 4, 75%–100%. The final score was calculated by multiplying the two primary scores. Final scores of 0–4 were defined as negative, and final scores of 8–12 as positive.

Flow cytometry

Cells were seeded in 6-well plates overnight to reach 70%–80% confluence. After the indicated treatments, the cells were digested using trypsin, washed twice with phosphate-buffered saline, and stained using the Annexin V-FITC/PI or Annexin V-633/PI Apoptosis Detection Kit (Dojindo) according to the manufacturer's instructions. Stained cells were analyzed using a FACSCelesta multicolor flow cytometer (Becton Dickinson, Franklin Lakes, NJ USA) and the data were processed using FlowJo software.

In situ proximity ligation assay (PLA) assay

The Duolink In Situ Red Kit (Sigma-Aldrich, St. Louis, MO, USA) was used to visualize direct protein-protein interactions in Panc1 cells. Briefly, cells were seeded onto 6-well plates overnight at 70%–80% confluence. The cells were washed twice with cold PBS and fixed with 4% paraformaldehyde for 15 min at room temperature. The fixed cells were then permeabilized with PBS containing 1% Triton X-100 for 30 min, subsequently blocked in Blocking Solution (Sigma-Aldrich) at 37°C for 1 h and incubated with primary antibodies (anti-N1ICD [CST, #4147]; anti-USP10 [CST,

#8501] and anti-N1DARP [generated and validated by Abcam]) overnight at 4°C. The next day, cells were washed twice with washing buffer, and incubated with PLA probes in a ratio of 1:4 in antibody diluent for 1 h at 37°C. Subsequently, the cells were incubated with the ligation solution at 37°C for 30 min and then with amplification solution at 37°C for 100 min. Duolink *in situ* mounting medium mixed with DAPI was added to the cells and incubated at room temperature for 15 min. Images were captured using a confocal microscope (FV1000; Olympus).

Preparation of fusion proteins and *in vitro* pulldown assay

Glutathione S-transferase (GST)-fused N1ICD was cloned into a pGEX-2T vector, which was then expressed in *Escherichia coli* and affinity-purified using glutathione-Sepharose (Pharmacia and Upjohn, London, UK). Ten micrograms of purified GST and GST-N1ICD fusion protein was mixed with Panc1 cell lysates transfected with 10 μg of N1DARP-Flag or USP10-Flag and incubated in binding buffer (0.8% BSA in PBS in the presence of the protease inhibitor mixture) overnight at 4°C under gentle rotation. Subsequently, the binding reaction was added to 30 μL of glutathione-Sepharose beads and mixed at 4°C for 2 hours. The beads were washed five times with binding buffer, resuspended in 50 μL of 2 × SDS-PAGE loading buffer, and resolved on a 10% gel. Protein bands were detected with anti-N1DARP or anti-USP10 antibodies by western blotting.

Biotin pull-down analysis was performed as previously described[3]. Biotin-labelled SAH-mAH2-5 and SAH-CTRL were generated using EZ-Link™ NHS-Biotin (Thermo Fisher Scientific). Biotinylated polypeptides were incubated with the PDAC-R organoid cell lysates at 37 °C for 30 min. Interacting protein complexes were isolated using streptavidin-conjugated Dynabeads (Thermo Fisher Scientific). The precipitated components were subjected to SDS–PAGE, followed by Coomassie blue staining.

Differentially expressed proteins were cut for mass spectrometry (LTQ Orbitrap XL).

Immunoprecipitation and Mass Spectrometry (MS)

Pancreatic cancer cells transfected with the indicated plasmids or lentiviruses were collected and lysed on ice for 10 min. Centrifugation was performed to obtain the supernatant, followed by incubation with the appropriate antibody and Protein A/G Plus-Agarose (Santa Cruz Biotechnology, Santa Cruz, CA, USA) at 4°C overnight. The immunocomplex was washed 4–6 times and boiled in 2×SDS sample buffer for 5 min. The coprecipitates were resolved by SDS-PAGE and blotted with specific antibodies. Bound proteins were dissolved in SDS sample buffer and analyzed by immunoblotting. For mass spectrometry (MS), Capan1 cells were treated with Flag-N1DARP for 48h and lysed. Whole cell lysates were extracted and immunoprecipitated with an anti-FLAG antibody using a Pierce CO-IP Kit (Thermo Fisher Scientific). The eluents were separated using SDS-PAGE, followed by Coomassie blue staining, as shown in Fig. 4C. The bands were extracted from the gel and subjected to LC-MS/MS sequencing and data analysis by QL Bio Biotechnology Co., Ltd. (Beijing, China). MS/MS data were searched against the human FASTA sequence from UniProt using an in-house Proteome Discoverer (Version PD1.4, Thermo Fisher Scientific). Peptides assigned only to a given protein group were considered unique.

Tumor xenograft assay

All animal experimental procedures were performed in compliance with the institutional ethical requirements and approved by the Shanghai Jiao Tong University School of Medicine Committee for the Use and Care of Animals. Nude male BALB/c mice (4–6 weeks old) were purchased from the

Chinese Academy of Sciences (Shanghai, China) and maintained in a specific pathogen-free facility. For the subcutaneously injected tumor model, pancreatic cell lines (Capan1 and Panc1) (1×10^7 cells/site) and patient-derived organoids (PDAC1 and PDAC2) (5×10^6 cells/site) transfected with the empty vector and N1DARP or *LINC00261* were injected subcutaneously into the right flank of each mouse. Tumor volumes were measured every 7 days for 35 days from the first injection using the formula: tumor volume (mm^3) = 1/2 ($a\times b^2$), in which "a" represents the longest longitudinal diameter, and "b" is the longest transverse diameter.

To assess the effect of SAH-mAH2-5 on survival in tumor-bearing mice, we established a pancreatic orthotopic implantation model by inoculating luciferase-labeled Capan1 cells (5.0×10^5) and four organoids (1.0×10^5) mixed with Matrigel into the pancreas of each mouse. After one week, SAH-CTRL/SAH-mAH2-5 (1/2/3/4/5 mg/kg) at various concentrations was intravenously injected weekly. *In vivo* imaging was performed to monitor the development of Capan1 cells and organoids in BALB/c nude mice treated with peptides using an IVIS Spectrum optical imaging system every week after inoculation.

To evaluate the toxicity of SAH-mAH2-5, healthy BALB/c nude mice (5 weeks) were intravenously administered SAH-CTRL or SAH-mAH2-5 (2 mg/kg) once a week for 4 weeks. Mice were then sacrificed, and some organs, including the pancreas, lungs, spleen, kidneys, liver, and colon, were extracted and HE stained to screen for morphological abnormalities. In addition, serum was collected for the biochemical analysis of alanine aminotransferase (ALT), aspartate aminotransferase (AST), blood urea nitrogen (BUN), and creatinine (Cr) using a Chemistry Analyzer TBA-40FR (Toshiba Medical System, Tokyo, Japan).

Chloroalkane Penetration Assay (CAPA)

Capan1 cells stably expressing Halo-GFP-Mito were seeded in 12-well plates. After 24 h, cells were washed with PBS and treated with chloroalkane-tagged peptides in phenol red-free Opti-MEM for 4 h. The medium was aspirated and the cells were washed with Opti-MEM for 15 min. The cells were then incubated with 5 μM HaloTag® TAMRA Ligand (Promega, Madison, WI, USA) in phenol red-free Opti-MEM for 15 min. After washing with PBS, the cells were analyzed using a fluorescence microplate reader or fluorescence microscope.

Metabolic stability of peptide

The sample solution consisted of 15 μl peptide solution (1 mM) and 585 μl Tris-HCl (pH = 7.4) buffer. Pepsin solution (4,500 U/ml, 8 μl) or proteinase K solution (4 ng/ml, 8 μl) was added to the sample solution and incubated at 25°C for indicated times. Peptide concentrations were determined by reverse-phase HPLC (Waters Symmetry 3.5 μm 4.6 × 150 mm C18) and detected by Exactive™ Plus Orbitrap (Thermo Fisher Scientific).

In vitro plasma stability assay

In vitro stability of peptides was assessed in rat plasma. The rat blood was collected by retroorbital puncture, and plasma was harvested after centrifugation (2,500×g, 5 min, 4°C) and stored at -80°C. The reactions were initiated by adding the test peptide to 100 μl of preheated plasma solution to yield a final concentration of 4 mM. The plasma solutions were incubated for indicated times, and then 200 μl of acetonitrile was added to deproteinize the plasma. The samples were subjected to vortex mixing for 1 min and centrifugation for 15 min (14,000 rpm, 4°C). The peptide concentration in the supernatant

was determined using reversed phase HPLC with electrospray ionization mass spectrometric detection.

Supplementary Note S1. Generation and modification of N1DARP-derived stapled peptide SAH-mAH2-5.

To evaluate the therapeutic potential of disrupting the N1ICD-USP10 interaction with N1DARP and define a more druggable target, we simulated the tertiary structure of N1DARP using I-TASSAR, a hierarchical server for protein structure prediction[4], and identified two α-helical peptides (AH1 and AH2) (Fig. S8A, left panel). Surface plasmon resonance (SPR) analysis showed that the purified AH2 peptide of NI1DARP had a higher affinity for the ANK domain (Fig. S8A, right panel). More precisely, we constructed and purified six subdomains with α-helix secondary structure of ANK, designated from ANK1 to ANK6, and SPR analysis demonstrated that AH2 was more prone to interact with ANK1 (Fig. S8B-S8D). To optimize the physicochemical properties of AH2, we first checked the amino acid sequence and found that Glu32, a negatively charged amino acid, might impinge on the permeability of cancer cells to AH2; therefore, we tried amino acid substitution of Glu32 by Gln32. SPR analysis showed a comparable affinity between ANK and AH2 or substituted AH2, which was designated as mAH2 (Fig. S8E). Next, to identify the indispensable amino acids mediating mAH2 and ANK interactions, we substituted each amino acid of mAH2 with Ala, followed by SPR analysis. The results showed that Tyr26, Leu28m, and Leu33 were essential amino acids for mAH2 and ANK interaction (Fig. S8E). Based on this result, we modified mAH2 with molecular staples inserted at five positions (*i*, *i* + 4, or *i* + 7) and denoted them as stapled α-helices (SAHs). SPR analysis showed that SAH-mAH2-5 had the highest affinity for ANK (Fig. S8F-S8H). Molecular docking using CABS-dock demonstrated that SAH-mAH2-5 interacted with ANK by embedding itself into the surface groove of ANK by

electrostatic attraction and hydrogen bonding (Fig. S8I). To measure the permeability of SAH-mAH2-5, we performed a chloroalkane penetration assay using Capan1 cells stably expressing Halo-GFP followed by fluorescence confocal microscopy (Fig. S8J), and observed that introduction of chloroalkane-linked SAH-mAH2-5 (cl-SAH-mAH2-5) resulted in a weaker cl-TAMRA signal than the introduction of chloroalkane and Pep2-linked mAH2 (cl-Pep2-mAH2) (Fig. S8K). Besides, CP50 (the concentration at which 50% peptide penetration was detected) of cl-SAH-mAH2-5 was more than twentyfold lower than that of cl-Pep2-mAH2 (Fig. S8L). Both assays suggested that SAH-mAH2-5 had better permeability and cytoplasmic localization than Pep2-mAH2. To detect physiochemical stability, mAH2, Pep2-mAH2, and SAH-mAH2-5 were incubated with protease K, pepsin, and mouse plasma, followed by liquid chromatography/mass spectrometry. SAH-mAH2-5 exhibited not only drastically enhanced resistance to proteases but also a prolonged half-life in plasma, suggesting a remarkable physiochemical stability of SAH-mAH2-5 (Fig. S8M-S8O).

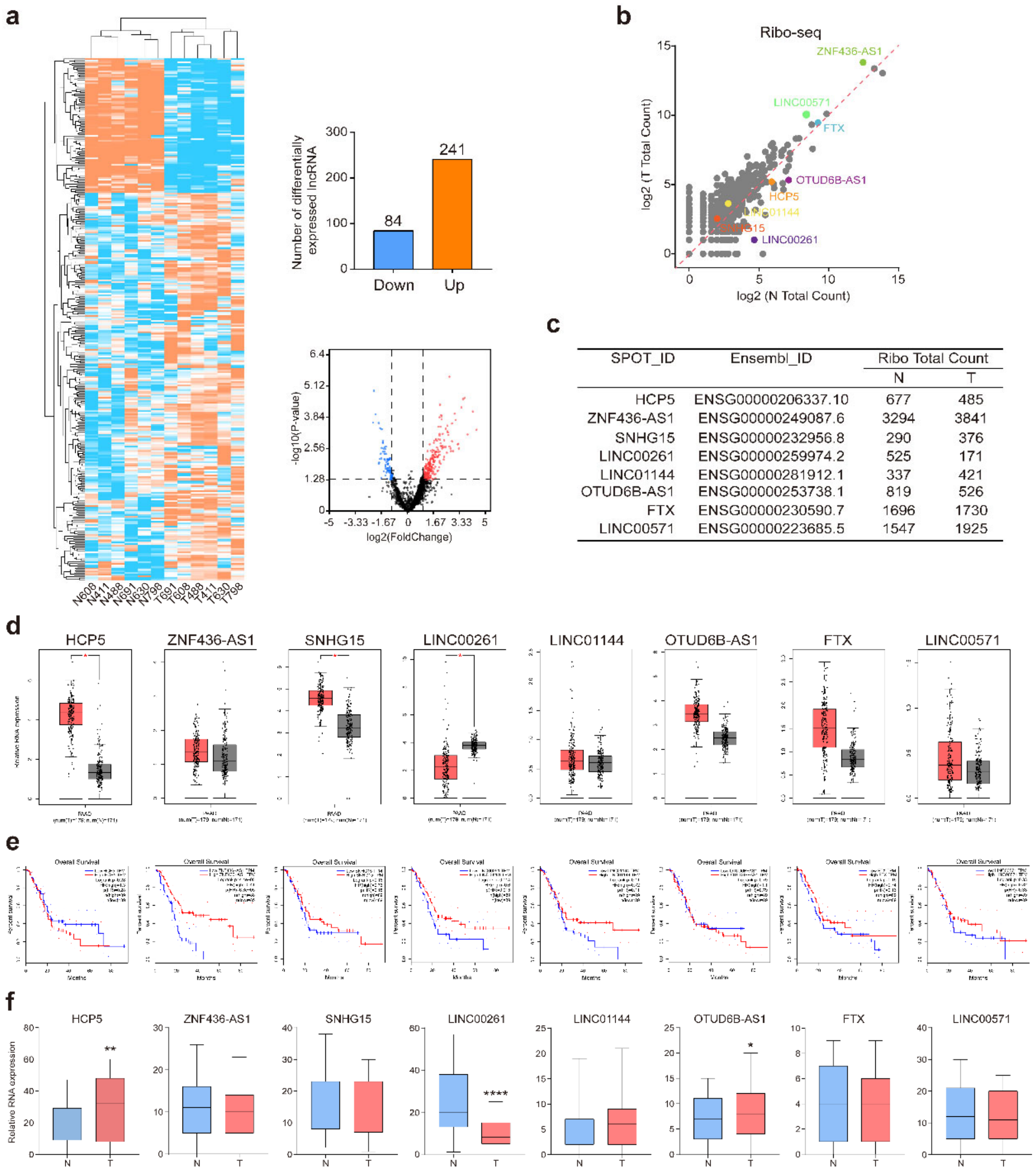

SPOT_ID	Ensembl_ID	Ribo Total Count	
		N	T
HCP5	ENSG00000206337.10	677	485
ZNF436-AS1	ENSG00000249087.6	3294	3841
SNHG15	ENSG00000232956.8	290	376
LINC00261	ENSG00000259974.2	525	171
LINC01144	ENSG00000281912.1	337	421
OTUD6B-AS1	ENSG00000253738.1	819	526
FTX	ENSG00000230590.7	1696	1730
LINC00571	ENSG00000223685.5	1547	1925

Supplementary Figure S1.

Profiling of expression and translation of lncRNAs in pancreatic cancer vs. normal pancreatic cells. (a) Left: Differentially expressed lncRNAs annotated in NONCODE in six pairs of pancreatic cancer cells and corresponding normal pancreatic cells. Upper right: Statistics of dysregulated lncRNAs. Lower right: Volcano plot of differentially expressed lncRNAs. (b) The translational status of lncRNAs annotated in NONCODE as measured by ribosome profiling in pancreatic cancer and normal pancreatic cells. (c) Information and junction reads of the eight overlapping lncRNAs that were

differentially expressed and translated. (d) Expression and (e) survival analyses using data from TCGA database. (f) Expression of the eight overlapping lncRNAs in 75 pairs of pancreatic cancer and normal pancreatic tissue samples. Data are presented as mean ± standard deviation of three independent experiments. *P < 0.05, **P < 0.01, ****P < 0.0001 by Student's t test (d, f); P < 0.05 was considered statistically significant by log-rank test (e).

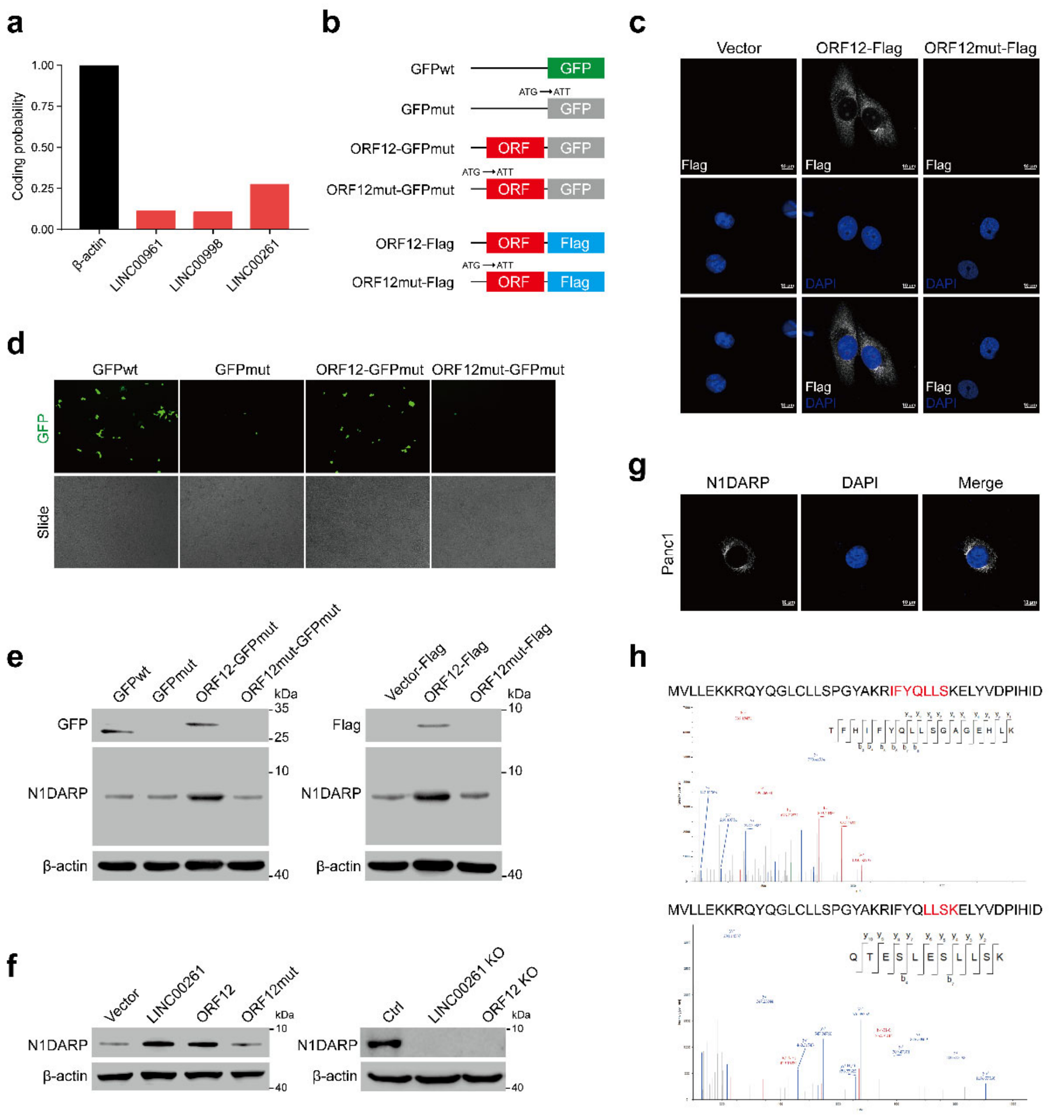

Supplementary Figure S2.

N1DARP exists in pancreatic cancer cells. (a) Coding potential of *LINC00261* and other translatable

lncRNAs evaluated by CPC2. (b) Diagram illustrating the construction of GFP or FLAG-tagged ORF12s and their corresponding mutants. (c) Immunofluorescence analysis detecting the expression status of FLAG-tagged ORF12 and ORF12 with mutated start codon in Panc1. (d) Fluorescence microscopy detecting the translation status of GFP-tagged ORF12 and its mutants in Panc1. (e) Detection of the expression of GFP or FLAG-tagged ORF12 wild type and start codon mutants using western blot in HEK293T. (f) The specificity of rabbit polyclonal antibody against N1DARP detected using western blot in Panc1. (g) The endogenous expression and subcellular location of N1DARP detected using immunofluorescence microscopy in Panc1. (h) The existence of N1DARP identified using mass spectrometry analysis using Panc1 transfected with Flag-ORF12. The data represent three independent experiments.

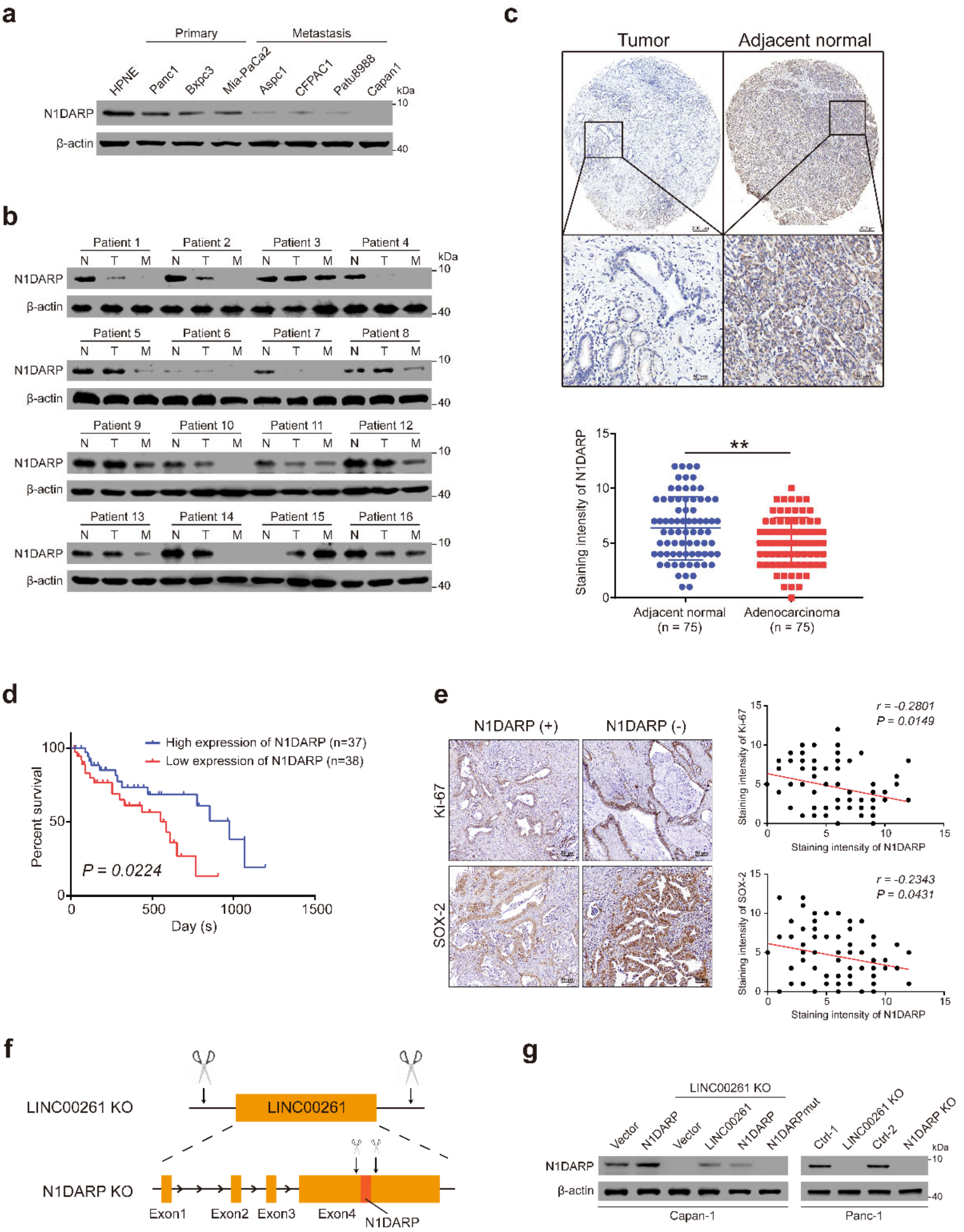

Supplementary Figure S3.

N1DARP expression is inhibited in pancreatic cancer and denotes better prognosis. (a) The expression level of N1DARP in pancreatic cancer cell lines from primary lesion or liver metastasis. (b) The expression of N1DARP in sixteen pairs of adjacent normal tissue, primary tissue, and liver metastasis obtained from sixteen metastatic pancreatic cancer patients. (c) Top: N1DARP expression in

tissue microarray from seventy-five pairs of pancreatic cancer and adjacent normal tissues. Bottom: quantified staining intensity of N1DARP in seventy-five pairs of pancreatic cancer and adjacent normal tissues. (d) The log-rank curve using follow-up data of seventy-five pancreatic cancer patients mentioned above divided by N1DARP expression in tissue microarray. (e) Left: correlation between the expression of N1DARP and Ki-67/SOX2 in seventy-five pancreatic cancer patients in tissue microarray. Right: linear regression using quantified staining intensity of N1DARP and Ki-67/SOX2. (f) Illustration of Crispr/Cas9-mediated LINC00261 and N1DARP knockout in pancreatic cancer cell and mouse model. (g) Confirmatory expression of N1DARP in Capan-1 with N1DARP overexpression or *LINC00261* knockout, and in Panc-1 with *LINC00261* and *N1DARP* knockout. The data are presented as the mean ± standard deviation of three independent experiments. **$P < 0.01$ by Student's t test (c); $P < 0.05$ was considered statistically significant by log-rank test (d); $P < 0.05$ was considered statistically significant by Pearson's correlation test (r, Pearson's correlation coefficient) (e).

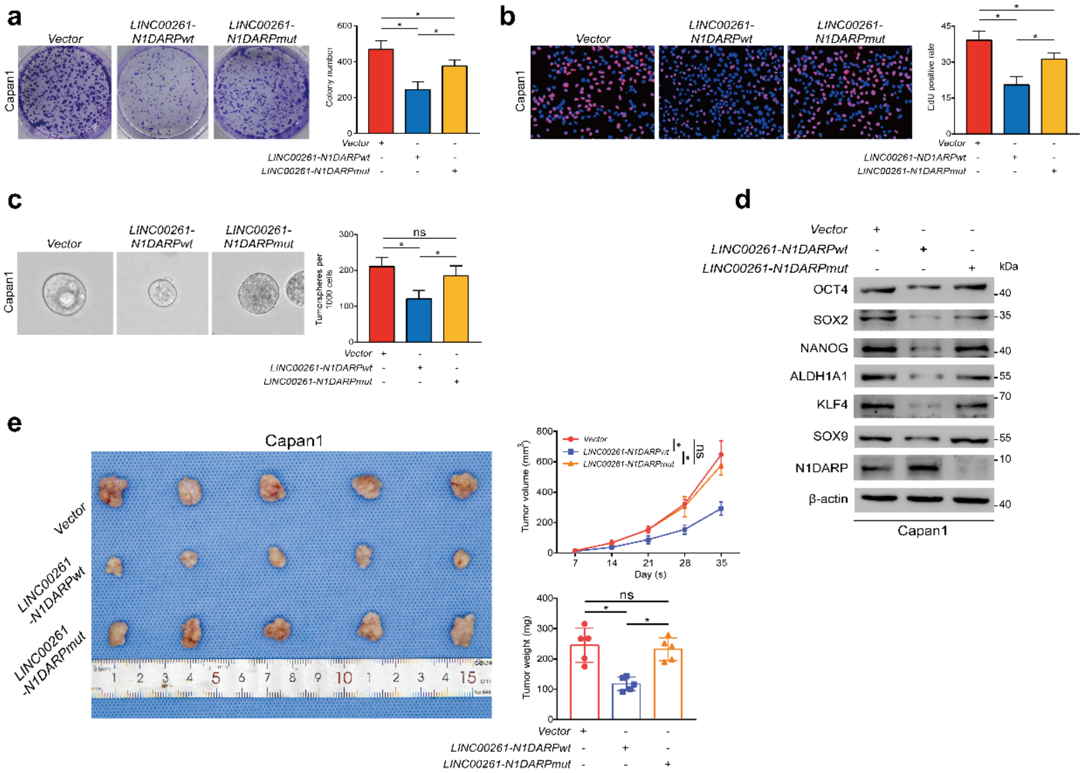

Supplementary Figure S4.

Supplementary Figure S4.

N1DARP is essential for LINC00261 tumor suppressive function. Cell proliferation detected by (a) colony formation and (b) EdU assay, and stem cell properties assessed by (c) sphere formation assay and (d) western blot using Capan1 transfected with *LINC00261* containing N1DARP wild type or start codon mutant. (e) Tumor volume and weight of subcutaneously injected Capan1 in nude mice transfected with *LINC00261* with N1DARP wild type or start codon mutant. The data are presented as the mean ± standard deviation of three independent experiments. ns, no significance; **P* < 0.05 by Student's t test (a-c, e for tumor weight); ns, no significance, **P* < 0.05 by one-way ANOVA (e for tumor volume).

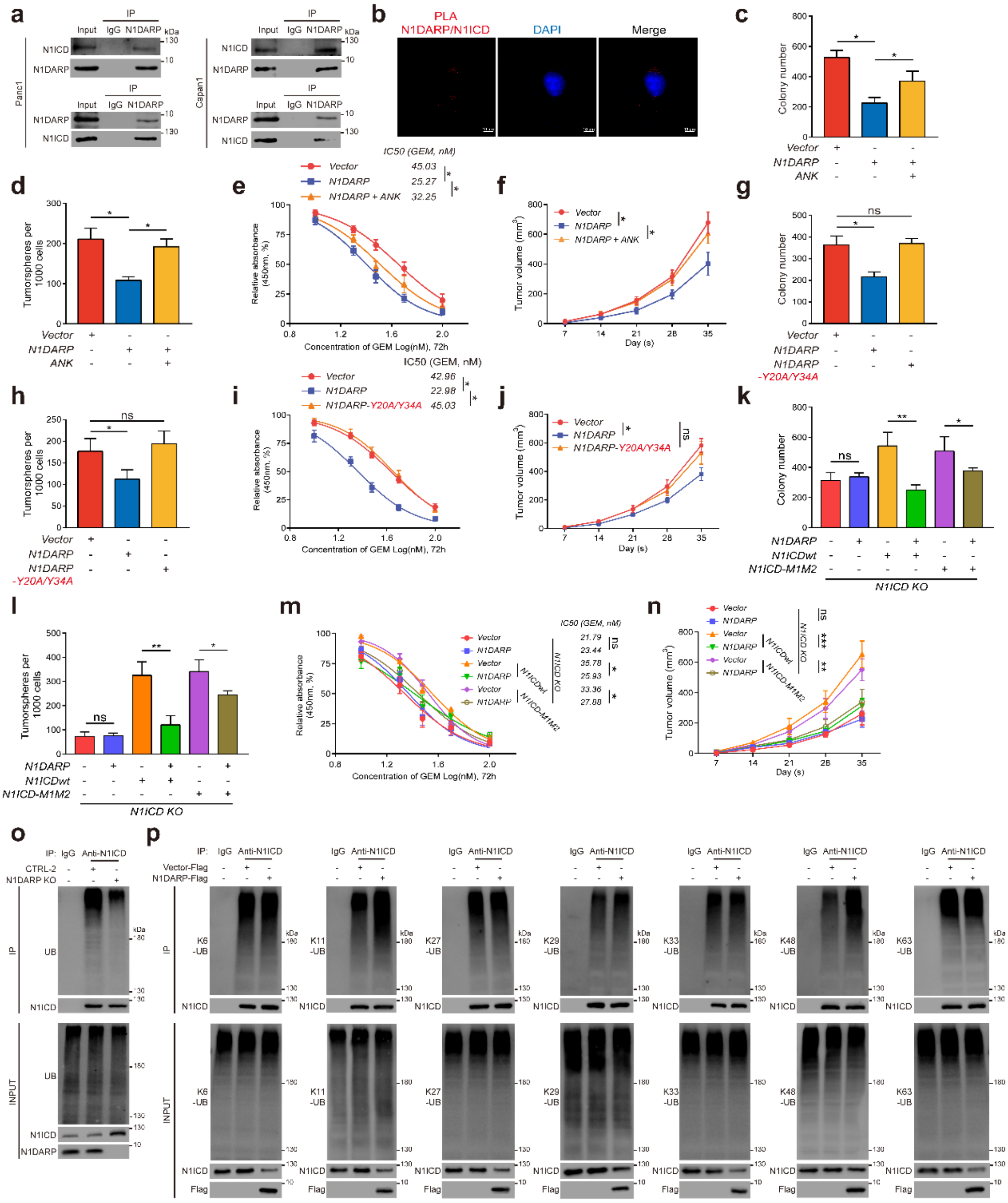

Supplementary Figure S5.

ANK repeat sequence serves as pivotal domain for N1DARP tumor suppressive function. (a) Co-IP followed by western blot verifying the N1DARP-N1ICD interaction in Panc1 and Capan-1. (b) PLA assay using Panc1 cell to detect the direct interaction between N1DARP and N1ICD visualized through confocal microscopy. Cell proliferation detected using (c) colony formation assay, (d) stemness measured by sphere formation assay, and (e) chemosensitivity to GEM assessed by IC50 assay using

Capan1 transfected with N1DARP with or without ANK domain of N1ICD. (f) Tumor volume of subcutaneously injected Capan1 transfected with N1DARP with or without ANK domain of N1ICD in nude mice. (g-i) Cell proliferation detected using colony formation assay, stem cell properties assessed using sphere formation assay and western blot, and chemosensitivity assessed using IC50 assay using Capan1 with N1DARP wild type or its Y20/Y34 mutant. (j) Tumor volume of subcutaneously injected Capan1 transfected with N1DARP wild type or its Y20/Y34 mutant in nude mice. (k-m) Cell proliferation detected using colony formation assay, stem cell properties assessed using sphere formation assay and western blot, and chemosensitivity assessed using IC50 assay using N1ICD knockout Capan1 transfected with N1DARP and wild type N1ICD or its M1M2 mutant. (n) Tumor volume of subcutaneously injected N1ICD knockout Capan1 transfected with N1DARP and wild type N1ICD or its M1M2 mutant in nude mice. (o) Total polyubiquitination level of N1ICD detected by western blot using Panc1 transfected with N1DARP knockout. (p) Specific polyubiquitination of N1ICD detected by co-IP followed by western blot using Capan1 with vector or N1DARP. The data are presented as the mean ± standard deviation of three independent experiments. ns, no significance; *$P < 0.05$ by Student's t test (c, d, g, h, k, l); ns, no significance, *$P < 0.05$, **$P < 0.01$, ***$P < 0.001$ by one-way ANOVA (e, f, i, j, m, n).

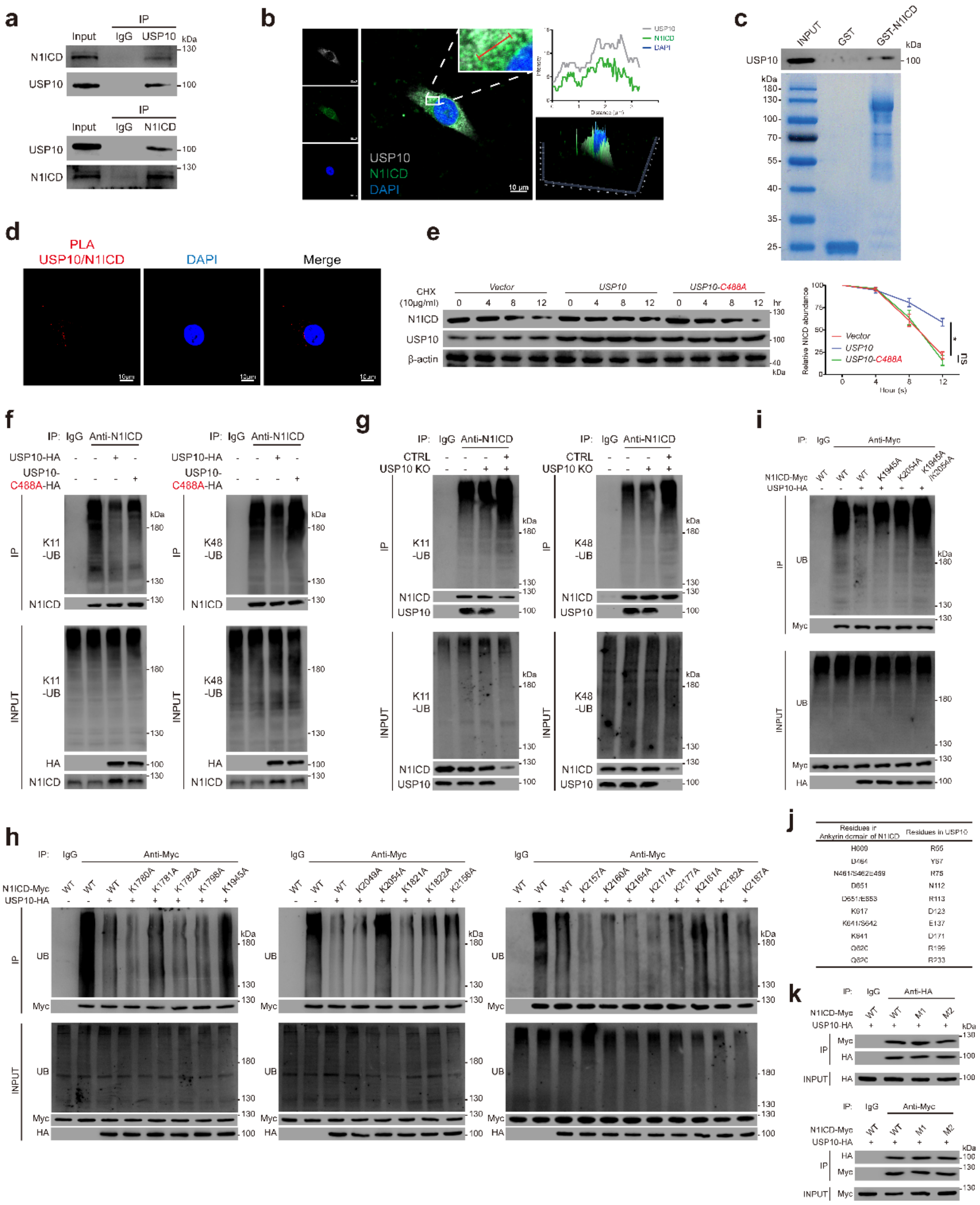

Supplementary Figure S6.

USP10 interacts with and promotes deubiquitination of N1ICD. (a) Co-ip followed by western blot verifying USP10-N1ICD interaction in Capan1. (b) Immunofluorescence confocal microscopy using Panc1 corroborating colocalization of USP10 and N1ICD in the cytoplasm by detecting simultaneously elevated fluorescence intensity of these two proteins. (c) *In vitro* pulldown assay using purified GST and GST-tagged N1ICD fusion protein incubated with Panc1 cell lysates, followed by western blot to

detect the direct interaction between USP10 and N1ICD. (d) PLA assay using Panc1 cell visualized by confocal microscopy to detect the direct interaction between USP10 and N1ICD. (e) Remaining N1ICD at indicated time detected by western blot in Capan1 with wild type or C488A functionally deficient mutant USP10 after treatment with CHX. K11- and K48-linked polyubiquitination level of N1ICD detected by co-IP and western blot using K11- or K48-linkage specific polyubiquitination antibody (f) in Capan1 transfected with wild type USP10 or its functionally deficient mutant (USP10-C488A), and (g) in Capan1 with USP10 knockout. (h) Specific Lys ubiquitination sites in ANK by USP10 predicted using NetChop 3.1 and validated using co-IP and western blot using Capan1 transfected with wild type N1ICD or predicted Lys mutant. (i) Polyubiquitination level of N1ICD detected by co-IP and western blot using Capan1 transfected with USP10, along with wild type, K1945A, K2054A or double sites mutant (K1945A/K2054A) of N1ICD. (j) Essential amino acids mediating USP10-ANK interaction predicted using ClusPro 2.0. (k) Co-IP and western blot using Capan1 transfected with USP10, as well as N1ICD wild type or M1 and M2 mutants, to detect USP10 and mutant N1ICD interaction. The data represent three independent experiments. ns, no significance; $^*P < 0.05$ by one-way ANOVA (e).

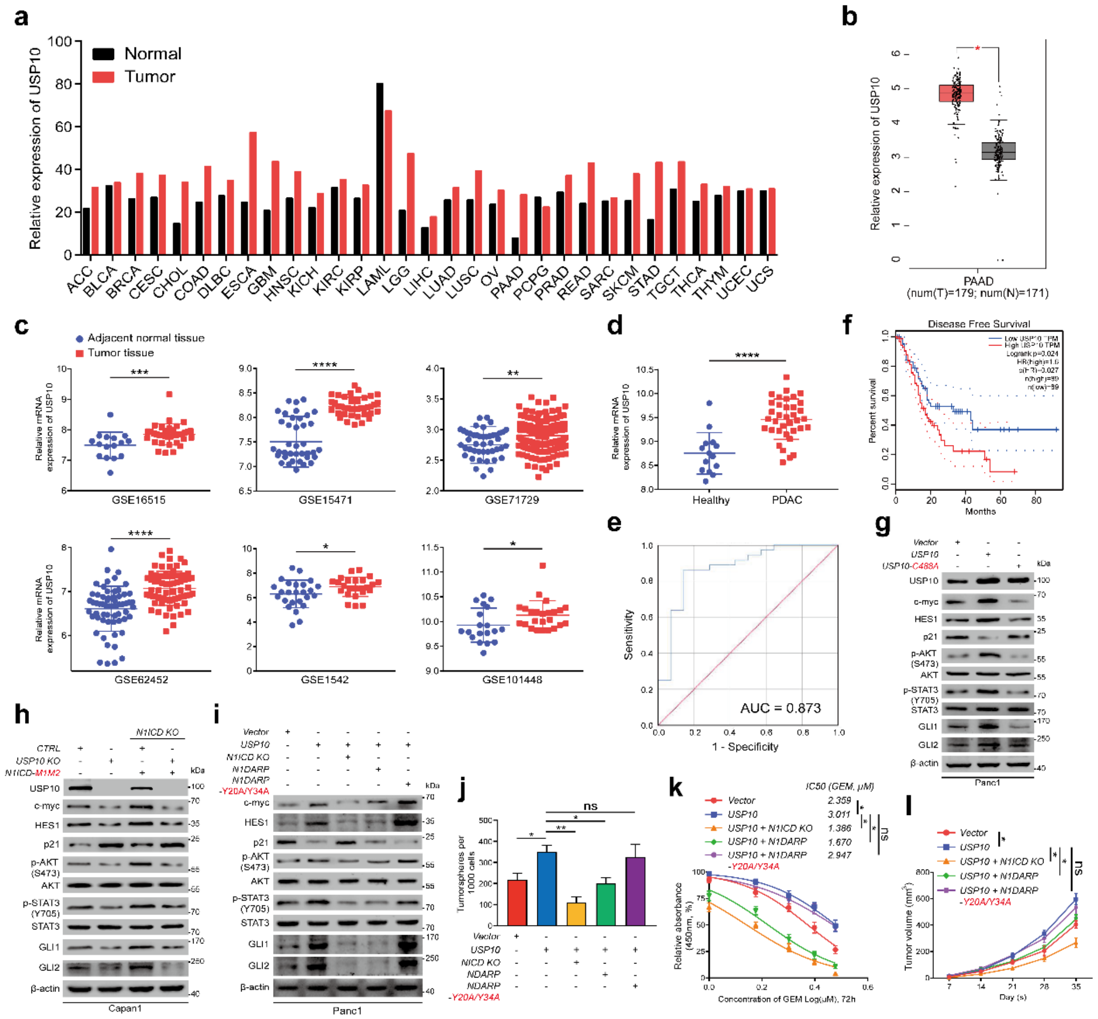

Supplementary Figure S7.

USP10 upregulation in pancreatic cancer promotes tumor progression and chemoresistance by deubiquitinating and stabilizing N1ICD. The expression of USP10 in adjacent normal tissue compared with tumor tissue in (a) pan-cancer and (b) pancreatic cancer using expression data from TCGA database. (c) The expression of USP10 in adjacent normal tissue compared with tumor tissue in pancreatic cancer using expression data from GEO datasets. (d) The expression of USP10 in serum comparing healthy people with cancer patients using expression data from GEO datasets. (e) The AUC curve generated from expression data of GSE15471. (f) The survival analysis of USP10 using follow-up data of pancreatic cancer patients from TCGA database. (g) Notch signaling and its crosstalk with other pathways detected using western blot using Panc1 transfected with wild type USP10 or its C488

functionally deficient mutant. (h) Notch signaling and its crosstalk with other pathways detected using western blot using Capan1 with N1ICD knockout and transfection with N1ICD-M1M2 mutant followed by USP10 knockout. (i) Notch signaling and its crosstalk with other pathways detected using western blot using Panc1 with USP10 overexpression followed by introduction of N1DARP wild type or Y20/Y34 mutant. (j) Tumor sphere formation, (k) chemosensitivity measured using IC50 assay, and (l) subcutaneously injected tumor volume using Panc1 with USP10 overexpression followed by introduction of N1DARP wild type or Y20/Y34 mutant. The data are presented as the mean ± standard deviation of three independent experiments. *$P < 0.05$, **$P < 0.01$, ***$P < 0.001$, ****$P < 0.0001$ by Student's t test (b, c, d, j); $P < 0.05$ was considered statistically significant by log-rank test (f); ns, no significance; *$P < 0.05$ by one-way ANOVA (k, l).

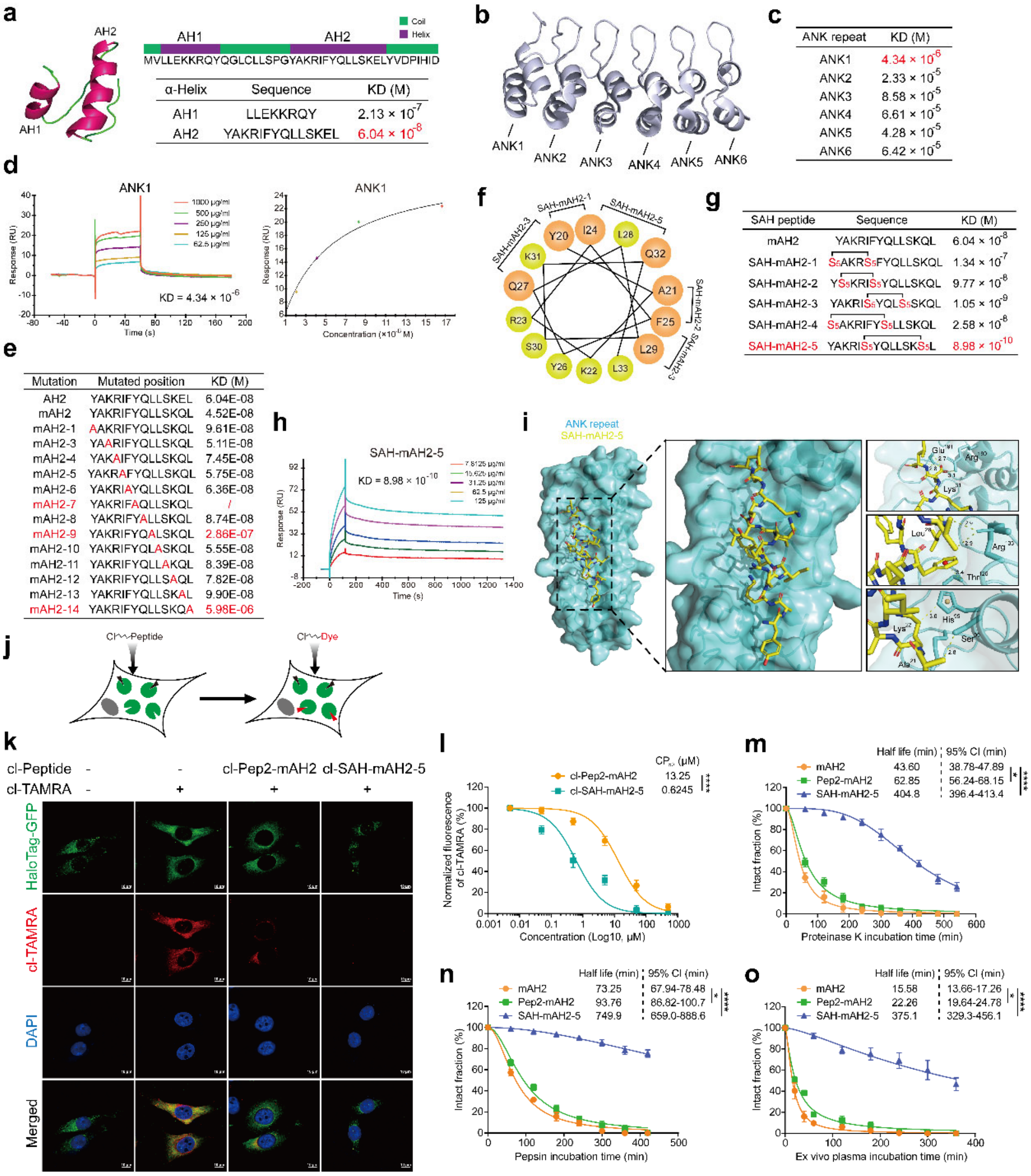

Supplementary Figure S8.

Generation and modification of N1DARP-derived stapled peptide SAH-mAH2-5. (a) Tertiary and secondary structure of N1DARP as predicted by I-TASSAR and the affinity of two α-helices (AH1 and AH2) for ANK domain measured by Surface plasmon resonance (SPR). (b) Six α-helix-containing regions of ANK domain obtained from PDB database. (c) Affinity of six α-helix containing regions to AH2 detected by SPR. (d) Kinetic association and disassociation between AH2 and ANK1 detected by SPR. (e) Affinity of modified AH2 (mAH2) for ANK domain with each amino acid substitution to Ala

detected by SPR. (f) Diagram of amino acids in mAH2 replaced by S5 for generation of stapled peptide. (g) Affinity of various stapled peptides for ANK domain as detected by SPR. (h) Kinetic association and disassociation between SAH-mAH2-5 and ANK domain as detected by SPR. (i) Molecular docking simulated by ClusPro 2.0 and presented by PyMOL showing SAH-mAH2-5 embedded in the surface groove of ANK through electrostatic attraction, hydrogen bonding, and proper conformation. (j) Schematic diagram of chloroalkane penetration assay (CAPA). (k) Confocal microscopy detecting the intensity of chloroalkane-linked TAMRA (cl-TAMRA) after incubation of cl-Pep2-mAH2 or SAH-mAH2-5 with HaloTag stably expressed Capan1 cells. (l) Average cl-TAMRA fluorescence intensity measured by confocal microscopy in HaloTag stably expressed Capan1 cells treated with different concentrations of cl-Pep2-mAH2 or SAH-mAH2-5. The physiochemical stability of SAH-mAH2-5 compared with mAH2 and Pep2-mAH2 incubated with (m) proteinase K, (n) pepsin, and (o) serum plasma of mice. The data are presented as the mean ± SD of three independent experiments. *$P < 0.05$, ****$P < 0.0001$ by one-way ANOVA (l-o).

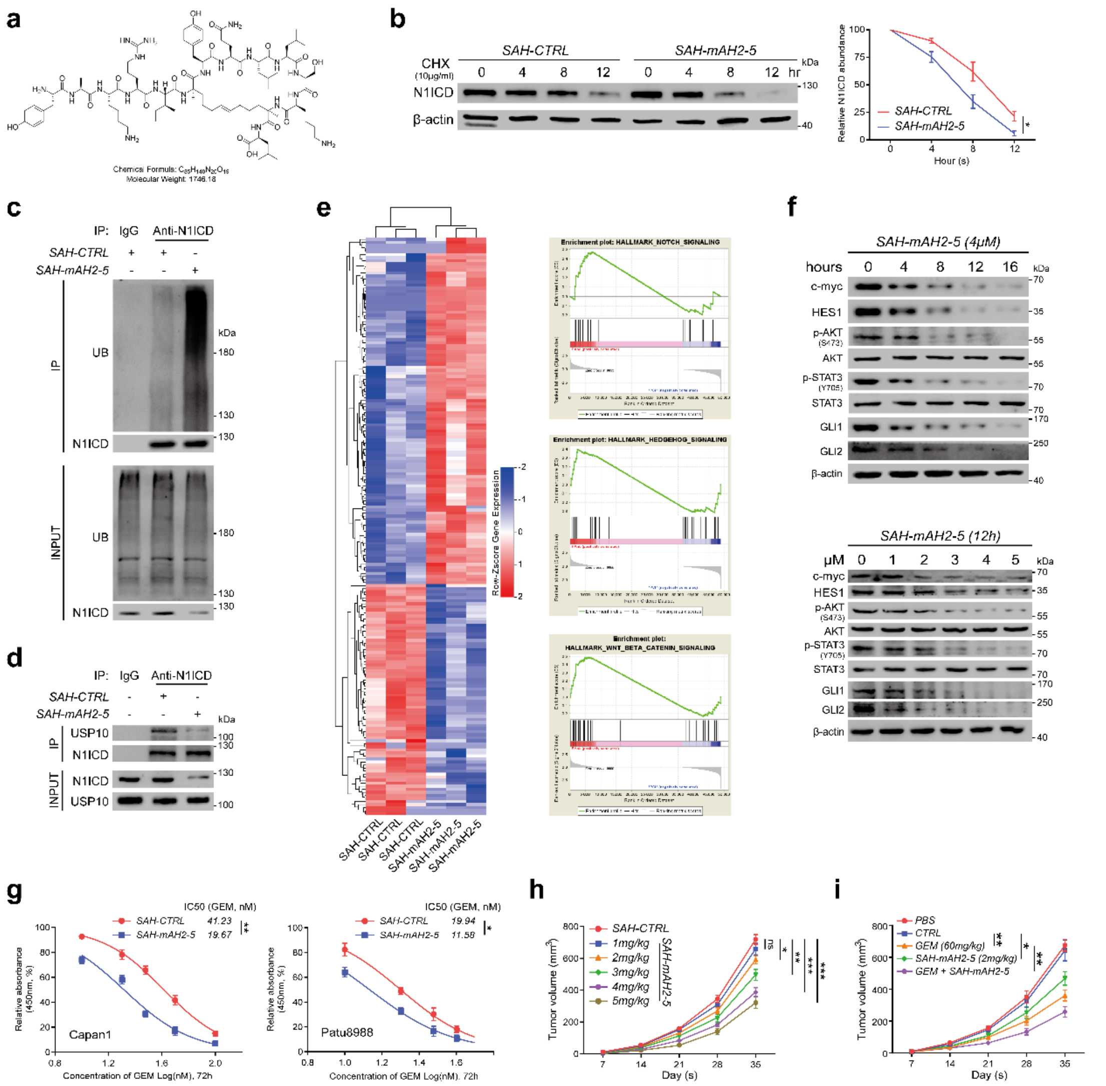

Supplementary Figure S9.

SAH-mAH2-5 suppressed tumor initiation and progression by disrupting USP10-N1ICD interaction. (a) The molecular structure of SAH-mAH2-5. (b) After treatment with CHX, remaining N1ICD at indicated time detected using western blot in Capan1 incubated with SAH-CTRL or SAH-mAH2-5. (c) Total ubiquitination level of N1ICD detected using western blot using Capan1 incubated with SAH-CTRL or SAH-mAH2-5. (d) The effect of SAH-mAH2-5 on the USP10-N1ICD interaction detected using western blot in Capan1. (e) Left: RNA sequencing analysis using Capan1 treated with SAH-CTRL or SAH-mAH2-5; Right: GSEA analysis of differentially expressed genes in RNA sequencing results. (f) The inhibition of Notch signaling and its crosstalk with other pathways detected

using western blot at indicated time or at indicated concentration using Capan1 incubated with SAH-mAH2-5. (g) Chemosensitivity measured by IC50 assay using Capan1 and Patu8988 with SAH-CTRL or SAH-mAH2-5. (h) Subcutaneous tumor volume in BALB/c nude mice inoculated with Capan1 and treated with SAH-CTRL or various concentrations of SAH-mAH2-5. (i) Subcutaneous tumor volume in BALB/c nude mice inoculated with Capan1 and treated with GEM, SAH-mAH2-5, or a combination of these two compounds. The data are presented as the mean ± standard deviation of three independent experiments. ns, no significance; *$P < 0.05$, **$P < 0.01$, ***$P < 0.001$ by one-way ANOVA (b, g-i).

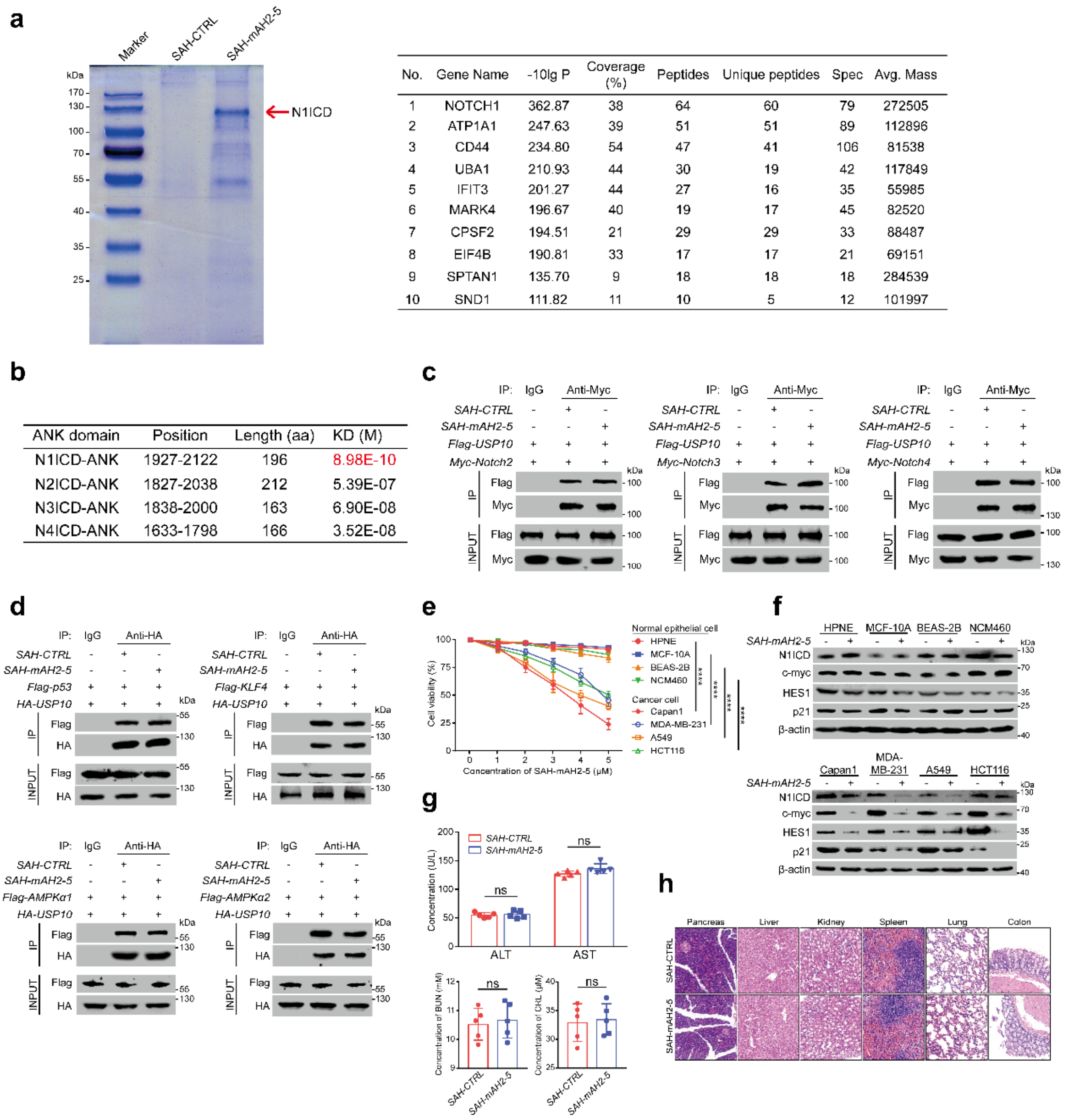

Supplementary Figure S10.

Off-target and toxic effects of SAH-mAH2-5. (a) Left: Biotin labeled pull-down assays of SAH-mAH2-5 in PDAC-R organoid followed by SDS–PAGE separation and Coomassie blue staining; Right: Top ten interactive proteins with biotinylated SAH-mAH2-5 determined using mass spectrometry**.** (b) Affinity of various ANK domain derived from Notch1, Notch2, Notch3, and Notch4 to SAH-mAH2-5 as detected by SPR. (c) The effect of SAH-mAH2-5 on the interaction of USP10 with other Notch receptors (Notch2, Notch3, and Notch4) in Capan1 detected using western blot. (d) The effect of SAH-mAH2-5 on the interaction of USP10 with other known substrates (p53, KLF4, and AMPK subunits) detected using western blot. (e) Cell viability detected using CCK8 assay of cancer and corresponding normal cell lines from the pancreas, breast, lung, and colon treated with different concentrations of SAH-mAH2-5. (f) The protein expression of N1ICD and its target gene in cell lines mentioned above with or without SAH-mAH2-5 incubation. (g) Serum concentration of ALT/AST and BUN/CRE in mice treated with SAH-CTRL or SAH-mAH2-5. (h) HE staining of the pancreas, liver, kidney, spleen, lung, and colon of mice treated with SAH-CTRL or SAH-mAH2-5. The data represent three independent experiments. ns, no significance by Student's t test (g); ****$P < 0.0001$ by one-way ANOVA (e).

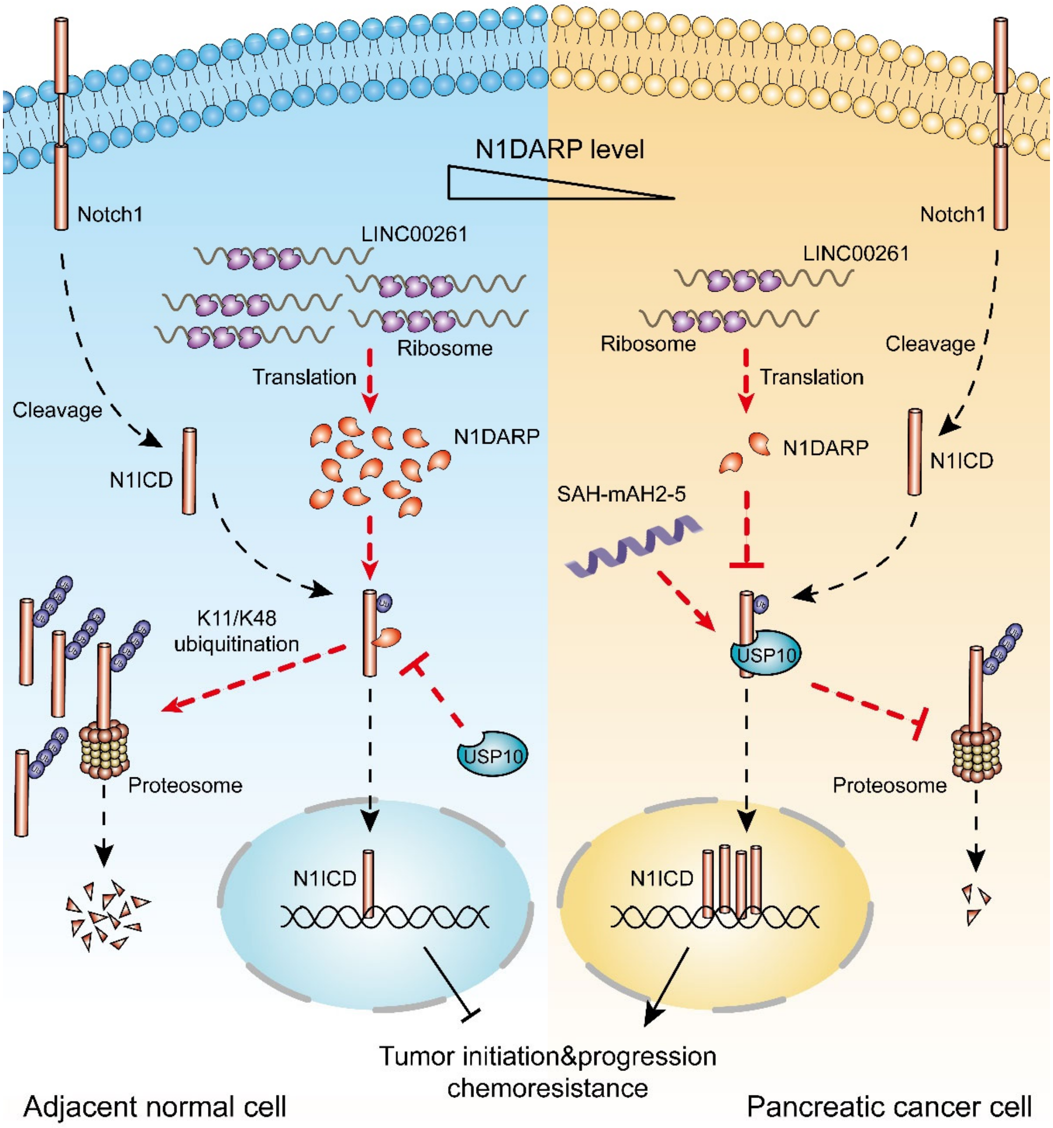

Supplementary Figure S11.

Graphical summary. N1DARP, encoded by LINC00261, is downregulated in pancreatic cancer and exerts tumor suppressive function by disrupting USP10-N1ICD interaction to promote the K11- and K48-linked polyubiquitination of N1ICD. SAH-mAH2-5, with a helical structure similar to N1DARP, interacted with and promoted the proteosome-mediated degradation of N1ICD.

Supplementary Table S1. The correlation between N1DARP expression and clinicopathological features of patients with pancreatic cancer

Characteristics		n=75	Expression of N1DARP		*P* value
			Low expression	High expression	
**Gender**	Male	42 (56.0%)	24 (57.1%)	18 (42.9%)	0.205
	Female	33 (44.0%)	14 (42.4%)	19 (57.6%)	
**Age(years)**	≥60	48 (64.0%)	20 (41.7%)	28 (58.3%)	0.247
	＜60	27 (36.0%)	15 (55.6%)	12 (44.4%)	
**Pathologic stage**	I	9 (12.0%)	3 (33.3%)	6 (66.7%)	0.033*
	II	24 (32.0%)	11 (45.8%)	13 (54.2%)	
	III+IV	42 (56.0%)	30 (71.4%)	12 (28.6%)	
**T stage**	T1+T2	22 (29.3%)	10 (45.5%)	12 (54.5%)	0.019*
	T3+T4	53 (70.7%)	39 (73.6%)	14 (26.4%)	
**N stage**	N0	20 (26.7%)	12 (60.0%)	8 (40.0%)	0.534
	N1	40 (53.3%)	18 (45.0%)	22 (55.0%)	
	N2	15 (20.0%)	8 (53.3%)	7 (46.7%)	
**M stage**	M0	57 (76.0%)	31 (54.4%)	26 (45.6%)	0.853
	M1	18 (24.0%)	10 (55.5%)	8 (44.5%)	

**P*<0.05 was considered to denote statistical significance.

Supplementary References

1. Na Li, *et al.* KISS-1 inhibits the proliferation and invasion of gastric carcinoma cells. *World J Gastroenterol.* 2012;18:1827-1833.
2. H Kato, *et al.* Involvement of RBP-J in biological functions of mouse Notch1 and its derivatives. *Development.* 1997;124:4133-4141.
3. W Wang, *et al.* HuR regulates p21 mRNA stabilization by UV light. *Mol Cell Biol.* 2000;20:760-769.
4. Jianyi Yang, *et al.* The I-TASSER Suite: protein structure and function prediction. *Nat Methods.* 2015;12:7-8.
